# Interaction of Vaccination and Reduction of Antibiotic Use Drives Unexpected Increase of Pneumococcal Meningitis

**DOI:** 10.1038/srep11293

**Published:** 2015-06-11

**Authors:** Matthieu Domenech de Cellès, Margarita Pons-Salort, Emmanuelle Varon, Marie-Anne Vibet, Caroline Ligier, Véronique Letort, Lulla Opatowski, Didier Guillemot

**Affiliations:** 1 Institut Pasteur, Unité de Pharmaco-Épidémiologie et Maladies Infectieuses, F–75015 Paris, France; 2 INSERM, U1181, F–75015 Paris, France; 3 Univ. Pierre et Marie Curie, Cellule Pasteur UPMC, F–75005 Paris, France; 4 Univ. Versailles Saint Quentin, UFR des Sciences de la Santé Simone-Veil, EA 4499, F–78180 Montigny–le-Bretonneux, France; 5 AP–HP, Hôpital Européen Georges-Pompidou, Laboratoire de Bactériologie, F–75015 Paris, France; 6 Centre National de Référence des Pneumocoques, F–75015 Paris, France; 7 École Centrale Paris, Laboratoire de Mathématiques Appliquées aux Systèmes, F–92290 Châtenay-Malabry, France; 8 AP–HP, Hôpital Raymond-Poincaré, Unité Fonctionnelle de Santé Publique, F–92380 Garches, France

## Abstract

Antibiotic-use policies may affect pneumococcal conjugate-vaccine effectiveness. The reported increase of pneumococcal meningitis from 2001 to 2009 in France, where a national campaign to reduce antibiotic use was implemented in parallel to the introduction of the 7-valent conjugate vaccine, provides unique data to assess these effects. We constructed a mechanistic pneumococcal transmission model and used likelihood to assess the ability of competing hypotheses to explain that increase. We find that a model integrating a fitness cost of penicillin resistance successfully explains the overall and age-stratified pattern of serotype replacement. By simulating counterfactual scenarios of public health interventions in France, we propose that this fitness cost caused a gradual and pernicious interaction between the two interventions by increasing the spread of nonvaccine, penicillin-susceptible strains. More generally, our results indicate that reductions of antibiotic use may counteract the benefits of conjugate vaccines introduced into countries with low vaccine-serotype coverages and high-resistance frequencies. Our findings highlight the key role of antibiotic use in vaccine-induced serotype replacement and suggest the need for more integrated approaches to control pneumococcal infections.

*Streptococcus pneumoniae* (the pneumococcus) is a Gram-positive bacterium that frequently colonizes the human nasopharynx, but can also invade other body sites to cause a broad spectrum of infections^1^. The pneumococcus imposes a high public health burden, accounting for an estimated 11% of all deaths in children <5 years old worldwide^2^. In the early 2000 s, pneumococcal conjugate vaccines (PCV) were licensed for young children, and were shown protective against carriage and infection by a limited number of serotypes that caused a high proportion of invasive diseases in the United States^3^. Because of the vaccine’s serotype specificity, however, serotype replacement following widespread vaccination caused early concern^4^. After the introduction of the 7-valent PCV (PCV7), two markedly different outcomes were observed. Several authors reported little serotype replacement and a sharp decline of invasive pneumococcal diseases in children <2 years old and, to a lesser extent, in other age groups not directly targeted by the vaccine, as a consequence of herd immunity^5^,^6^,^7^. In other regions, however, considerable serotype replacement was observed^8^, and data even indicated small or no net benefits of vaccination—e.g., Spain^9^, and France^10^. Although several mechanisms have been proposed (e.g., secular trends in serotype distribution, changes in diagnostic practices), the causes of this variability of outcomes remain unclear^11^. Antibiotic use has been suggested to affect serotype replacement^12^,^13^, but rigorous evaluation of this hypothesis is required.

The reported increase of pneumococcal meningitis (PM) in France, where a national campaign to reduce antibiotic use was implemented in parallel to PCV7 introduction, provides unique data to examine these effects. Here we combined these incidence data and national data on antibiotic use and vaccination to construct a mechanistic model of pneumococcal transmission. Using likelihood, we aimed to assess the ability of competing hypotheses to explain that unexpected increase.

## Results

### Unexpected dynamics of pneumococcal meningitis in France

**Figure 1:**
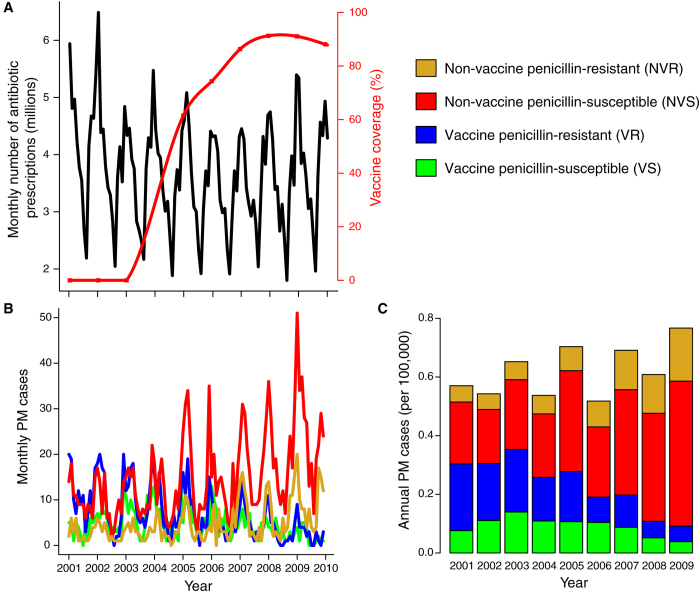
Public health interventions and dynamics of pneumococcal meningitis (PM) in France. (**A**) Monthly number of antibiotic prescriptions (beta-lactams and macrolides) in France (black curve), and 7-valent pneumococcal conjugate-vaccine (PCV7) 2-dose coverage in children <2 years old (red curve). Antibiotic data are from the NHI system; vaccine coverage data are yearly estimates from a permanent 1/97 NHI sample (red squares, text S2 and Table S2) (**B**) Monthly and (**C)** annual PM cases (per 100,000 population) reported to the National Reference Center for Pneumococci. The biennial pattern apparent in **C** is caused by different notification rates between odd and even years; we corrected for this effect in our analyses (Supplementary Text S1 and Text S2).

As shown in Fig. 1A, PCV7 was introduced in 2003 for children <2 years old, in whom the seven targeted vaccine-serotypes were responsible for 70% of invasive pneumococcal diseases in France at that time. PCV7, initially recommended for children at high risk of infection, was then universally recommended for children <2 years old in 2006; vaccine coverage gradually increased from 2003 and exceeded 80% by 2006. Concurrently, a national campaign to reduce antibiotic use was launched at the end of 2002, and achieved substantial prescription reduction over the ensuing 5 years^14^. Throughout this period, the French National Reference Center for Pneumococci (NRCP) collected PM-incidence data classified by serotype and antibiotic susceptibility. For simplicity, we grouped the serotypes according to inclusion in PCV7 (vaccine or nonvaccine serotype) and penicillin susceptibility (penicillin-susceptible with MIC ≤ 0.06 mg/L, or penicillin-resistant with MIC > 0.06 mg/L).

The data presented in Fig. 1B-1C reveal shifts of the serotypes causing PM and demonstrate substantial serotype replacement following PCV7 introduction. Before 2003, most PM were caused by vaccine-serotype penicillin-resistant (VR) strains and nonvaccine-serotype penicillin-susceptible (NVS) strains. Thereafter, VR-PM gradually decreased following PCV7 introduction, concomitant with a dramatic NVS-PM increase and a less pronounced NVR-PM increase (mostly caused by serotype 19A, Supplementary Fig. S7). Overall, a ~70% decrease of vaccine-serotype PM was observed from 2001–2003 to 2008–2009, outweighed by a ~120% rise of nonvaccine-serotype PM; this pattern resulted in a ~20% PM increase during this period^15^. Comparable trends were observed in young children and old adults (Supplementary Fig. S1), suggesting a common serotype-replacement mechanism. During the study period, the serotypes 19F, 14, and 23 were the most common vaccine serotypes; the serotypes 19A, 3, and 7F, which have been identified as major non-PCV7 serotypes in other countries^7^,^16^,^17^,^18^, were the most common nonvaccine serotypes. Notably, there was little indication of substantial trends in these serotypes prior to the introduction of PCV7 (data not shown).

### Mechanistic model and inference framework

**Figure 2:**
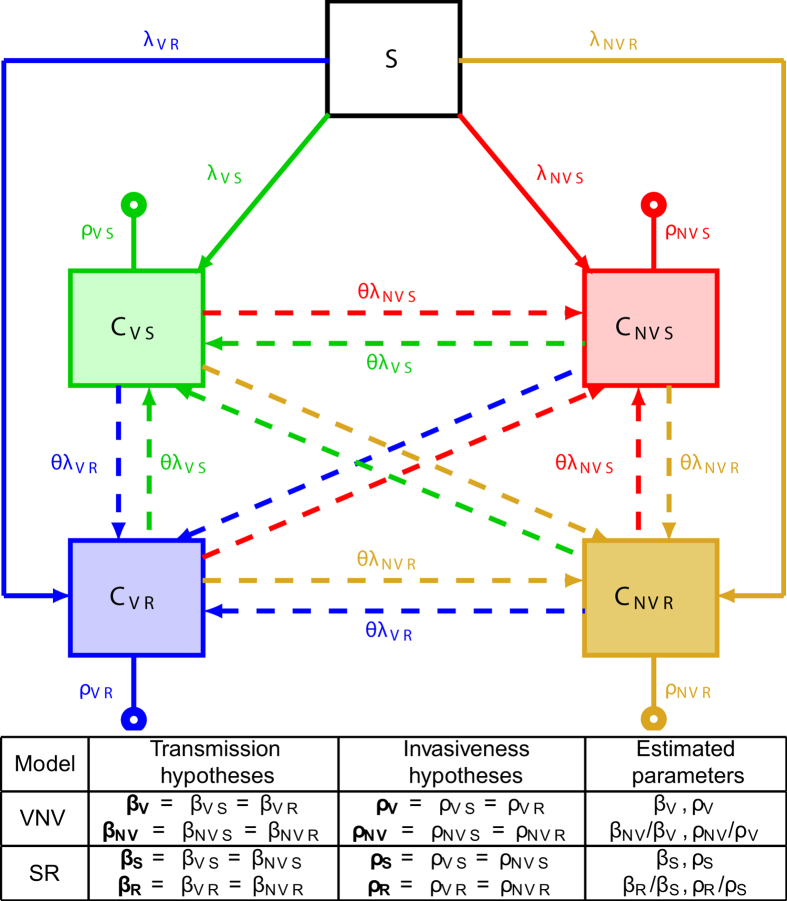
General model structure and derivation of the vaccine/nonvaccine (VNV) and susceptible/resistant (SR) models. (**Top**) Simplified model diagram. The population was divided according to carriage status: *S*, susceptibles; *C*_*VS*_, carriers of a vaccine-serotype, penicillin susceptible (VS) strain; *C*_*VR*_, carriers of a vaccine-serotype, penicillin-resistant (VR) strain; *C*_*NVS*_, carriers of a nonvaccine-serotype, penicillin-susceptible (NVS) strain; *C*_*NVR*_, carriers of a nonvaccine-serotype, penicillin-resistant (NVR) strain. The per-susceptible rate of pneumococcal acquisition, λ_X_, is serotype-dependent. Serotype-specific transmission rates β_X_ (where λ_X_ = β_X_*C*_X_/*N*) are assumed constant over time. Carriers of a given serotype can acquire another serotype (super-acquisition) at a rate reduced by a factor 1 − θ compared with noncarriers. Carriers develop PM at a serotype-dependent rate ρ_X_, which is assumed to be seasonal. In the full model, the population is further stratified according to vaccination and antibiotic exposure status, so that this simplified diagram is repeated 4 times (individuals unexposed to antibiotics and unvaccinated, exposed and unvaccinated, unexposed and vaccinated, exposed and vaccinated). All model details are given in Supplementary Text S1. (**Bottom**) The table summarizes the simplifying transmission and invasiveness hypotheses that define the VNV and SR models. In the VNV model, strain’s transmissibility and invasiveness differ between vaccine and nonvaccine serotypes; in the SR model, strain’s transmissibility and invasiveness differ between susceptible and resistant serotypes.

To understand these paradoxical trends, we constructed a mechanistic, population-based model of pneumococcal transmission and PM infection (Fig. 2 and Methods). The model was structured according to carriage, vaccination and antibiotic-exposure statuses. Detailed data on vaccination and antibiotic exposure were obtained from the National Health Insurance (NHI) system and included as covariates in the model. We hypothesized that biological differences between pneumococcal serotypes might have caused the observed PM dynamics in France. Specifically, we evaluated support for two classes of hypotheses represented by two different models (Fig. 2). The Vaccine/NonVaccine (VNV) model explored transmissibility or invasiveness differences between vaccine and nonvaccine serotypes; the Susceptible/Resistant (SR) model addressed transmissibility or invasiveness differences between penicillin-susceptible and penicillin-resistant strains. Both models integrated additional hypotheses on between-strain competition (defined as the relative rate of acquisition in carriers vs. noncarriers, Fig. 2) and PCV7 efficacy on carriage (defined as the reduction in the acquisition risk of vaccine-serotypes in vaccinees^19^), reflected in two estimated parameters. We parameterized both models by defining transmissibility and invasiveness ratios (Fig. 2), which were estimated from the monthly incidence data (Fig. 1B) using a state-of-the-art likelihood-based, sequential Monte-Carlo method allowing for measurement error, non-stationarity, and inclusion of covariates^20^. All model details are given in the Methods and in text S1 of the Supplementary Information.

### Fitness cost of penicillin resistance

The estimation results were unequivocal: the SR model outperformed the VNV model by a large margin (ΔAIC = 22, Supplementary Table S5). The SR-model parameter estimates revealed lower transmissibility (β_R_/β_S_ = 0.95, 99% CI: (0.95–0.96)) and lower invasiveness of penicillin-resistant strains (ρ_R_/ρ_S_ = 0.78, 99% CI: (0.54–0.93))—that is, a fitness cost of penicillin resistance. The estimates also indicated moderate between-strain competition for carriage (relative risk of acquisition in carriers vs. noncarriers: θ = 0.82, 99% CI: (0.60–1)).

**Figure 3:**
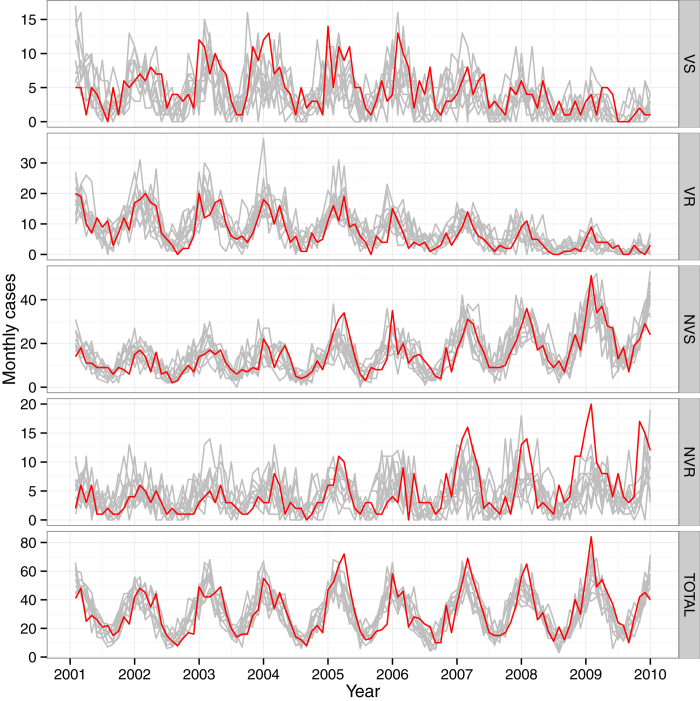
Model fit to data. For each strain, the observed numbers of PM (red curve), and the results of 10 stochastic runs of the SR model (thin grey lines) are shown. For completeness, the total number of PM (panel TOTAL) is also given; this series was not used for statistical inference. Note the different *y*-axis for each graph. VS: vaccine-serotype penicillin-susceptible; VR: vaccine-serotype, penicillin-resistant; NVS: nonvaccine-serotype penicillin-susceptible; NVR: nonvaccine-serotype penicillin-resistant.

The SR model provided good agreement with data (Fig. 3). The associated *R*^*2*^ values were 0.68 and 0.69 respectively, for the VR- and the NVS-PM series, which represented most of the PM occurring during the study period. As a result, the *R*^*2*^ associated with the whole PM series was 0.68. The largest discrepancy between model and data occurred for NVR PM. Notably, trends for the NVR-PM series were largely dominated by penicillin-resistant 19A, which increased steadily throughout the study period. This increase caused the resistance frequency among nonvaccine serotypes to remain relatively constant, while that frequency decreased among vaccine serotypes. To better understand how the dynamics of serotype 19A may have affected our results, we also performed additional estimations with a subset of data, from which we removed serotype-19A PM. These estimations indicated independent dynamics for this serotype, possibly because fitness cost was absent (Supplementary Text S3).

In agreement with carriage studies in French children^21^,^22^, the SR model predicted extensive replacement in carriage, with a gradual decrease of vaccine serotypes from 2004 and a concomitant increase of nonvaccine serotypes, particularly associated with penicillin-susceptible strains (Supplementary Fig. S6). Notably, although the model did not include a seasonal transmission term, it predicted a small seasonality in carriage. This pattern was caused by seasonal variations in antibiotic use and the fitness cost of resistance, which led to an increased circulation of penicillin-susceptible strains and a lower resistance frequency during summer months^23^.

To further assess the predictive power of the SR model, we separated the data into a training period (years 2001–2007) and a testing period (years 2008–2009) to perform out-of-fit (i.e., out-of-sample) predictions (Supplementary Fig. S10). This had little impact on parameter estimates (β_R_/β_S_ = 0.95, ρ_R_/ρ_S_ = 0.76), and the SR model had high predictive power for the most prevalent types (vaccine-type, penicillin-resistant VR and nonvaccine-type, penicillin-susceptible NVS) and overall.

### Sensitivity analyses

To assess the robustness of the SR model estimates, we conducted extensive sensitivity analyses. First, we varied the values of fixed model parameters and performed the estimations as before. For all the parameters tested, the estimates varied little (overall range of estimates: β_R_/β_S_ in 0.90–0.98 and ρ_R_/ρ_S_ in 0.64–0.90); crucially, the estimates always highlighted the existence of the fitness costs (Supplementary Table S6).

Second, because individual serotypes may differ in fitness, we tested how removing data from a single serotype affected our estimates (Supplementary Table S7). To do this, we identified in the French data the 3 most prevalent vaccine serotypes (19F, 14, and 23F, by decreasing order) and nonvaccine serotypes (19A, 3, and 7F, by decreasing order). We then created 6 new data sets by removing the data for each of these 6 serotypes, and we performed the estimations as before (that is, as we did for serotype 19A, cf above). While the estimated fitness cost on invasiveness remained <1 after removing any of serotype 14, 19F, 23F, and 19A (overall range of estimates: 0.23–0.95), this was not the case after removing serotype 3 (range: 0.5–1.2) or serotype 7F (range: 0.75–1.5). By contrast, the estimated fitness cost on transmission remained remarkably robust after removing any of these 6 serotypes (overall range: 0.95–0.96). Thus, although the estimated fitness cost on invasiveness appeared less robust, our estimate of the fitness cost on transmission, which drives the dynamics of replacement in carriage, remained unaltered.

### Model extension to include age structure

Because pneumococcal epidemiology varies by age, we considered an extended version of our model to include a simple age structure: [0, 3) y, [3, 16) y, and ≥16 y^24^. To do this, we extracted age-specific antibiotic use data and used data from the POLYMOD study to fix age-specific contact rates^25^; this extension also allowed us to consider age-specific durations of carriage^26^. Although we did not attempt to perform parameter estimation on this extended model, we found that a fitness cost on transmission remarkably close to our original estimate (0.945 instead of 0.954) explained qualitatively well the age-stratified incidence data in France (Supplementary Fig. S9). In particular, this model was able to qualitatively reproduce the rebound in pneumococcal meningitis in children aged [0, 3) y, the fast decrease of V-type PM concomitant with the strong replacement by NV-type (mostly penicillin-susceptible) PM in all age groups, and the overall increase in all age groups.

### Counterfactual scenarios of public health interventions

**Figure 4:**
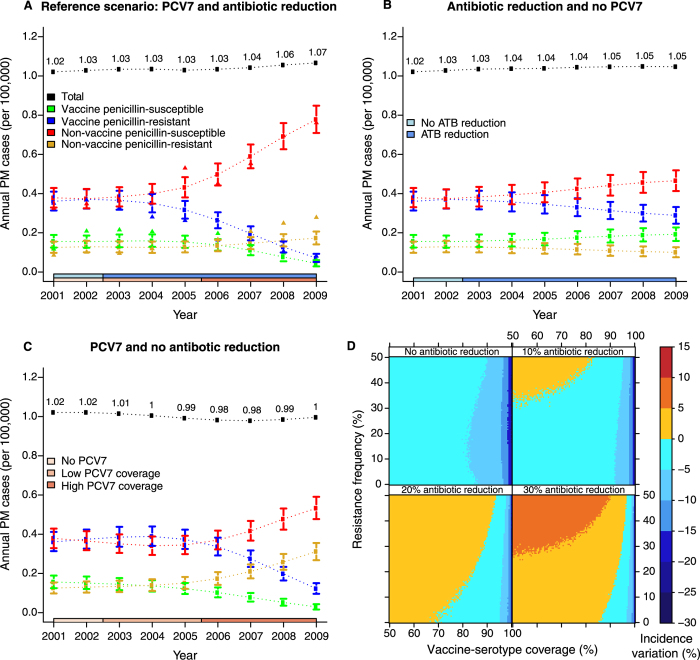
Simulated counterfactual scenarios of interventions in France and expected benefits of vaccination in various settings. (**A–C**) Model predictions according to intervention scenarios in France, 2001–2009: (**A**) Reference scenario with two interventions, (**B**) no PCV7 and antibiotic reduction, (**C**) PCV7 and no antibiotic reduction. The curves represent mean predicted annual cases (per 100,000 population) and the 95% prediction intervals for each type (green: VS, blue: VR, red: NVS, yellow: NVR) and for the total number of PM (black), obtained from 1,000 stochastic simulations of the best (SR) model with parameters fixed at their maximum likelihood values. In **A**–**C**, to avoid the biennial notification pattern, the simulated number of PM is represented before applying the observation model. In (**A**), the triangles represent the observed annual PM-incidence data from Fig. 1C, corrected for this biennial notification pattern (that is, divided by the reporting probability for each year). (**D**) Effects of vaccination and reduced antibiotic use. We simulated the effects of introducing a conjugate vaccine and reducing antibiotic use, while varying the initial vaccine-serotype coverage (proportion of PM caused by vaccine serotypes, *x*-axis) and the initial resistance frequency (*y*-axis). Here, we assume that, after a 2-year period without intervention, the two interventions are implemented simultaneously, and result in 100% vaccine coverage (all scenarios) and 0% (top left), 10% (top right), 20% (bottom left), or 30% (bottom right) reduction of antibiotic use. The colors represent the mean predicted variations of PM incidence (in %) 5 years after the implementation of the two interventions. Negative values correspond to decreases, while positive values correspond to increases.

Using the simple, non age-structured model, we tried to assess the relative contributions of PCV7 and antibiotic use to PM incidence during 2001–2009 by simulating the SR model for alternative scenarios of public health interventions in France (Fig. 4A–C). In a single-intervention–simulated scenario with no PCV7 and reduced antibiotic use (Fig. 4B), penicillin-susceptible PM continued to increase by the end of 2002, concurrent with a steady diminution of penicillin-resistant PM. Because of the higher transmissibility and invasiveness of penicillin-susceptible strains, these variations resulted in an overall, though moderate, PM rise during 2001–2009.

The single-intervention–simulated scenario with PCV7 and no antibiotic reduction displayed more complex dynamics, with higher strain replacement (Fig. 4C). Here, PCV7 introduction promptly interrupted the VR-PM increase caused by unrestricted antibiotic use; a sharp decline was predicted after 2004, causing a transient, overall PM reduction. With increasing vaccine coverage, however, PCV7 progressively conferred a selective advantage to nonvaccine strains, particularly more transmissible and more invasive NVS strains. Although this effect was delayed (starting after 2005), it reversed the overall PM decrease and caused an increase after 2007. Thus, this scenario highlighted two opposing PCV7 effects: quick reduction of vaccine serotypes, followed by a delayed, but more pronounced expansion of nonvaccine serotypes, particularly NVS serotypes. Crucially, the latter effect occurred because of the high resistance frequency and elevated prevalence of nonvaccine serotypes at PCV7 introduction.

Comparing these two scenarios with the reconstructed PM dynamics under the reference scenario (Fig. 4A) provides insight into the mechanisms of strain replacement in France. Results from the single-intervention scenario with no PCV7 suggested that the reduced antibiotic use mainly accounted for the 2003–2004 dynamics, with a small NVS-PM increase concomitant with a small VR-PM decrease. Akin to the single-intervention scenario with PCV7, PCV7 introduction then engendered the prompt VR-PM decline and a progressive NVS-PM rise. Because of the higher NVS-PM prevalence at PCV7 introduction, the two effects canceled each other out until 2006, resulting in a relatively constant total PM incidence during the period of low PCV7 coverage. After 2006, the NVS-PM rise drove an overall PM increase, qualitatively similar to that predicted in the single-intervention scenario with PCV7. Quantitatively, however, the NVS increase with the combined interventions (+120% from 2001–2002 to 2008–2009, Fig. 4A) exceeded the sum of the predicted increases of the two single-intervention scenarios (+28%, Fig. 4B and +36%, Fig. 4C). These results suggest an unexpected interaction between PCV7 and antibiotic-use reduction in enhancing the spread of NVS pneumococci, which, we propose, eventually caused the observed PM increase from 2006 to 2010.

### Interaction of vaccination and antibiotic-use reduction

Our scenario analysis suggests that, in addition to the synergistic interaction of the two interventions, the initially low vaccine-serotype coverage (that is, the proportion of PM caused by vaccine serotypes) and high resistance frequency were critical for high VR-strain replacement by NVS strains. Because those two quantities may vary widely among countries, we attempted to determine their impact on the expected benefits of vaccination with PCVs. To do so, we simulated a range of public health scenarios with PCV introduction and different reductions of antibiotic use (0 to 30%), while varying initial vaccine-serotype coverage and resistance frequency (Fig. 4D). In the scenarios with no antibiotic-use reduction, our model consistently predicted lower PM incidences, ranging from 0% to 30%, with reductions being higher for higher prevaccine vaccine-serotype coverage, regardless of the resistance frequency. In the scenarios with antibiotic-use reductions, however, the model predicted that low vaccine-serotype coverage and high antibiotic-resistance frequency could lead to a higher PM incidence, similar to the French experience. This impact was more pronounced for greater antibiotic reductions, indicating increasing interaction between the two interventions, consistent with our interpretation of the data in France. These outcomes suggest that public health interventions aimed at lowering pneumococcal resistance might counteract the benefits of vaccination with PCVs.

## Discussion

The difficulty of interpreting PM data from France, because of the gradual vaccine introduction and the concomitant campaign to lower antibiotic use, has been reported before^11^. Indeed, our findings indicate a complex association between the two interventions: the PCV7 effect gradually combined with the impact of antibiotic-use reduction to amplify NVS-pneumococcus replacement to finally cause the PM increase. That interpretation was only possible here because of our method, i.e., a mathematical model that integrated comprehensive data on vaccine and antibiotic use and that was confronted to detailed PM-incidence data using statistical inference. Previous studies highlighted a variety of mechanisms responsible for serotype replacement after vaccination, e.g., competition between vaccine and nonvaccine serotypes^27^,^28^,^29^, capsular switch^30^, invasiveness of nonvaccine serotypes^11^, or high pneumococcal antigenic diversity^4^,^31^. However, most of those studies focused on differences between vaccine and nonvaccine serotypes as the main forces driving serotype replacement postvaccination. Our findings also suggest the key roles of antibiotic use and competition between susceptible and resistant strains in modulating the effects of conjugate vaccines. While the heterogeneity of pneumococcal epidemiology worldwide precludes unequivocal recommendations, our findings have two potentially notable public health implications. First, under certain conditions (low vaccine-serotype coverage and high resistance frequency), initiating concomitantly vaccination and antibiotic-use reduction programs can lead to unexpected trends. Second, circulating serotypes and susceptibility to antibiotics, in addition to crude incidence estimates, should be carefully monitored after the rollout of vaccination.

Our results indicate lower transmissibility and lower invasiveness of penicillin-resistant pneumococci—that is, a fitness cost of resistance^32^. Determining the existence of this fitness cost, and, if so, its extent has been a central question in the epidemiology of pneumococcal resistance^33^. Previous evidence for a lower transmissibility of resistant pneumococci came, first, from laboratory studies that demonstrated a lower growth rate of resistant pneumococci in competition experiments *in vitro*^34^ or in mice^35^; and, second, from epidemiological studies that documented a rapid decrease of resistance after a reduction of antibiotic use^23^,^36^. Our estimate of the epidemic fitness cost is also consistent with that of previous modeling studies that used statistical inference on infection^37^ or carriage^38^,^39^ data. Regarding the impact of resistance on invasiveness, laboratory studies indicated a relationship between beta-lactam resistance and loss of virulence in mice^40^,^41^. Such a relationship was also suggested by epidemiological data, which documented a lower frequency of resistance in invasive isolates vs. noninvasive isolates^42^,^43^.

For scenarios with no antibiotic-use reduction, our model consistently predicted reductions of PM incidences, ranging from 0% to 30%, with reductions being higher for higher prevaccine vaccine-serotype coverage, regardless of the resistance frequency. Those findings might qualitatively explain the trends reported in countries that introduced PCV7 but, to our knowledge, did not lower antibiotic use^7^,^16^,^17^,^18^. While a systematic comparison across countries is beyond the scope of this study, we note that our model makes a testable prediction, supported by some epidemiological studies^23^,^36^. All else being equal, countries with high antibiotic use should have higher resistance but, paradoxically, lower infection rates than countries with low antibiotic use. Despite likely difficulties (e.g., confounding factors, variations in reporting across countries), testing this prediction will undoubtedly prove useful to confirm our findings.

Our model ignores several complexities associated with pneumococcal epidemiology. First, our model was not age-structured, even though children <2 years old, targeted by PCV7, play a large part in pneumococcal transmission and are at higher risk of developing PM. Notably, PCV7 serotype coverage was initially higher in this population, with ~70% of PM caused by vaccine serotypes in 2001 (Supplementary Fig. S1), in contrast to the ~50% value in the general population that was used in our model. In that age class, the PCV7 effect was initially more pronounced but was followed by a rebound in 2005–2006 that cancelled the benefit of vaccination by 2008–2009 (Supplementary Fig. S1 and refs 10,44,45). This rebound, despite higher vaccine coverage in 2005–2006, was previously difficult to account for, but fits our general interpretation. Again, the PCV7 effect initially contained the predominant VR PM, but, combined with reduced antibiotic-use and the fitness costs of resistance, gradually favored strong replacement by NVS PM (+260% from 2001–2002 to 2008–2009 versus +120% in the total population). Simulations from the extended age-structured model confirmed this interpretation (Supplementary Fig. S9); in addition, carriage studies in French young children provided evidence of a similar pattern in carriage^22^,^46^, which was also well-reproduced by our model’s carriage predictions (Supplementary Text S3 and Fig. S6). Therefore, our model also provides, at least qualitatively, a plausible explanation of the reported trends in this age class. Second, previous studies showed that pneumococcal serotypes differ in fitness^26^,^47^. For simplicity, we did not consider such differences in our model, and we grouped serotypes according to inclusion in PCV7 and antibiotic susceptibility. We note, however, that comparable models have successfully been applied to study serotype replacement after vaccination^27^,^28^,^48^,^49^,^50^. Finally, we did not consider multiple carriage, which might be frequent for pneumococcus^51^,^52^. Rather, we allowed for neutral super-acquisition between serotypes in the model, so that any carrier could acquire another strain at any given time, as in other modeling studies^53^,^54^.

Public health interventions used to limit the spread of infectious diseases are often responsible for side effects, or externalities, which can either be deleterious or advantageous^55^. Bacterial resistance is a well-known and extensively studied negative externality associated with antibiotic use, and previous studies highlighted the doubtless benefits of reducing antibiotic use to reduce the spread of resistant strains and to increase the fraction of treatable infections^56^,^57^. Our findings merely suggest that interventions aimed at lowering pneumococcus antibiotic-resistance might also engender negative side effects, linked to more infections caused by antibiotic-susceptible strains. Although further studies will be required to confirm our findings, we conclude that this possible pneumococcus-resistance dilemma could become a key public health question.

## Methods

### Pneumococcal meningitis data

Monthly PM-incidence data were obtained from the French National Center for Pneumococci (NRCP). Briefly, NRCP is a passive surveillance system that collects pneumococcal infection and carriage data through the Regional Pneumococcus Observatories, a network of 400 laboratories covering all regions of France. Each PM isolate was analyzed to determine its penicillin susceptibility and serotype, using standard procedures^58^. The data were then decomposed according to inclusion in the 7-valent pneumococcal conjugate vaccine (PCV7, vaccine serotypes, V, or nonvaccine serotypes, NV) and penicillin resistance (penicillin-susceptible strains, S, with MIC ≤ 0.06 mg/L and penicillin-resistant strains, R, with MIC > 0.06 mg/L). To assess the exhaustiveness of NRCP data, we compared NRCP numbers with the French Medicalization Program of Information System, a database that records all hospitalizations with a PM diagnosis (Supplementary Text S2). This comparison did not reveal any trend in the notification rate to NRCP; there were, however, systematic differences in notification rates between odd years (mean 65%, range 62–68%) and even years (mean 53%, range 49–58%). These differences were taken into account in the model (Supplementary Text S1).

### Antibiotic- and vaccine-use data

In France, the National Health Insurance (NHI, general fund) refunds medical care provided by physicians in private practice, community clinics, and hospitals. Data on antibiotic prescriptions—beta-lactams and macrolides—were extracted from the NHI database and corresponded to ambulatory prescriptions reimbursed by the two main NIH agencies (CNAM–TS and RSI, covering 85% of the French population). Because antibiotics are prescription drugs in France, these data are nearly exhaustive. Vaccine coverage in children <2 years old was obtained from yearly estimates for a permanent 1/97 sample of the NHI general fund (Supplementary Text S2 and Table S2).

### Demographic data

Demographic data in France, i.e., population size, birth rate and death rate, were available from the French National Institute of Statistics and Economic Studies (INSEE, http://www.insee.fr/en/; date of access: 01/03/2013).

### Model formulation and parameterization

All models were formulated as discrete, stochastic systems and simulated using Gillespie’s τ-leap algorithm with a time step of 1 day. The models were fitted to monthly PM-incidence data using the maximum iterated filtering algorithm, a likelihood-based, sequential Monte-Carlo method allowing for measurement error, non-stationarity, and inclusion of covariates^20^. All analyses were performed with the pomp package (http://pomp.r-forge.r-project.org/), operating in the R environment. The profile likelihood was used to compute approximate 99% confidence intervals^59^. All model details are given in the Supplementary Text S1.

## Additional Information

**How to cite this article**: Domenech de Cellès, M. *et al.* Interaction of Vaccination and Reduction of Antibiotic Use Drives Unexpected Increase of Pneumococcal Meningitis. *Sci. Rep.* **5**, 11293; doi: 10.1038/srep11293 (2015).

## Supplementary Material

Supplementary Information
