# Supplementary material for: Interaction of Vaccination and Reduction of Antibiotic Use Drives Unexpected Increase of Pneumococcal Meningitis
Source: Sci Rep. 2015 Jun 11;5:11293. doi: 10.1038/srep11293 (PMC4462765; doi:10.1038/srep11293)
Supplement: Supplementary Information [file srep11293-s1.pdf]

# Supplementary Information to: Interaction of Vaccination and Reduction of Antibiotic Use Drives Unexpected Increase of Pneumococcal Meningitis

Matthieu Domenech de Cellès, Margarita Pons-Salort, Emmanuelle Varon, Marie-Anne Vibet, Caroline Ligier, Véronique Letort, Lulla Opatowski, Didier Guillemot

List of abbreviations: PM, pneumococcal meningitis; VS: vaccine-serotype penicillin-susceptible; VR: vaccine-serotype, penicillin-resistant; NVS: nonvaccine-serotype penicillin-susceptible; NVR: nonvaccine-serotype penicillin-resistant; NRCP: French National Reference Center for Pneumococci; NHI: French National Health Insurance; INSEE: French National Institute of Statistics and Economic Studies; MPIS, Medicalization Program of Information System.

## Text S1. Supplementary Materials and Methods

### Mathematical model

**Basic structure.** We formulated a mechanistic, population-based model of pneumococcus dynamics in France. Because nasopharyngeal pneumococcus carriage precedes infection[1], we built a susceptible–infected–susceptible (SIS)-like compartmental model of pneumococcal carriage, in which carriers were at risk of developing pneumococcal meningitis (PM). The model integrated antibiotic exposure, vaccine coverage, and demography of the French population. The population was structured according to carriage status (susceptible/carrier), vaccination status (vaccinated/not vaccinated) and antibiotic exposure status (exposed/unexposed). For simplicity, we only considered two levels of penicillin susceptibility for carried strains: penicillin-susceptible strains, S, with MIC<0.06 mg/L and penicillin-resistant strains, R, with MIC≥0.06 mg/L. We also distinguished between PCV7-targeted serotypes (vaccine-serotype strains, V) and PCV7-

untargeted serotypes (nonvaccine-serotype strains, NV). Therefore, we considered four carriage statuses (Fig. 2, Main Text): carriers of a vaccine-serotype, penicillin-susceptible strain (VS); carriers of a vaccine-serotype, penicillin-nonsusceptible strain (VR); carriers of nonvaccine-serotype, penicillin-susceptible strain (NVS); and carriers of nonvaccine-serotype, penicillin-nonsusceptible strain (NVR).

**Between-strain competition.** Between-strain competition for carriage was previously suggested for pneumococcus[2]. To include it, we assumed that any strain-A carrier could acquire another strain B upon contact with a B-strain carrier, with B replacing A (super-acquisition). We also assumed that between-strain competition was independent of the carried strain[3,4]; this assumption gave a single competition parameter, θ, defined as the relative rate of acquisition in carriers versus noncarriers (Fig. 2, Main Text). Multiple-strain carriage was not allowed in the model.

**PM seasonality.** PM-incidence data showed strong seasonality (Fig. 1, Main Text), as do invasive pneumococcal diseases[5]. Because previous studies reported little seasonality of pneumococcal carriage[6,7], we incorporated seasonality into the PM-infection rate and not the transmission rate[8]. Therefore, the time-dependent, strain-specific infection rate, i.e., the rate at which carriers of a given strain develop PM, was modeled as:

$$\rho_{X,t} = \rho_X(1 + \hat{\rho}\cos(2\pi(t + \phi_\rho)))$$

where $\rho_X$ is the mean infection rate for strain X (*a priori* strain-dependent), $\hat{\rho}$ the amplitude of seasonality (assumed independent of the carried strain), the time (in years), and $\phi_\rho$ the phase, controlling the time-location peak. The parameter $\phi_\rho$ was estimated by fitting a simple sinusoidal function to each PM-incidence time-series; this procedure yielded a common estimated value, $\phi_\rho$=0.95, which corresponded to a PM peak at time $1 - \phi_\rho = 0.05$ in January.

**Initial conditions and demography.** The model was initialized using the French National Reference Center for Pneumococci (NRCP) 2001 data on the 1818 isolates collected that year. That collection was composed of 1052 strains isolated from invasive infections in adults ≥16 years old and 766 strains isolated from invasive infections and acute otitis media in children <16 years old[9]. We assumed that these data reflected the 2001 distribution of pneumococcal carriage in France, where NVS pneumococci (33% of isolates) and VR pneumococci (39% of isolates) were the most frequent. Reinforcing the validity of this assumption, we found similar values in a separate analysis in which we estimated initial conditions (data not shown). Demography was included in the model through a birth rate (0.0126/year) and a death rate (0.0086/year), assumed constant throughout the study period.

**Vaccine-related hypotheses.** Vaccinees were defined as children having received 2 doses of PCV7. The time-dependent proportion of vaccinated children (or vaccine coverage), v(*t*), was interpolated from yearly estimates provided by the National Health Insurance (NHI) permanent 1/97 sample (Fig. 1 Main Text and SI, Text S2) and was included as a covariate in the model. According to the French vaccine schedule, children receive 2 doses at age 2 and 4 months; for simplicity, however, we assumed that children were vaccinated immediately at birth. We assumed that vaccinees were completely protected against infection by a vaccine-serotype[10], but only partially protected against carriage by a vaccine-serotype. According to previous evidence[11], the PCV7 effect on carriage was modeled as a reduced acquisition risk of vaccine-serotypes in vaccinees by a factor $\sigma_V$[11]. This parameter was estimated in our analysis. We also assumed that PCV7 did not cross-protect against nonvaccine-serotypes.

**Antibiotic-related hypotheses.** The time-dependent, per capita antibiotic-exposure rate, $\tau(t)$, was interpolated from NHI monthly reimbursement data (Fig. 1, Main Text), and included as a

covariate in the model. Because carriage is typically asymptomatic, we supposed that all individuals were equally exposed to antibiotics, irrespective of their carriage status. We considered the following classes of antibiotics: beta-lactams (penicillins and cephalosporins) and macrolides, which represent 75–80% of antibiotic prescriptions in France[12]. Although we did not consider class-specific antibiotic exposure, we sought to reproduce those differences by computing a weighted average of each class's effect. Such a calculation might introduce bias if the relative frequency of each antibiotic class varies over time; as Fig. S2 shows, however, these relative frequencies varied little in France over 2001–2009, despite an overall reduction of antibiotic use[12]. The relative frequencies of each antibiotic class were 44% for penicillins, 28% for cephalosporins and 28% for macrolides. There are distinct mechanisms by which antibiotics can select for pneumococcal resistance in the population[13]. Here, we considered two mechanisms by which antibiotics favor pneumococcal resistance. First, antibiotics were supposed to enhance the clearance of carried pneumococci. This effect was modeled as an additional clearance rate under antibiotic treatment, $\omega(1-\sigma_S)$ for penicillin-susceptible strains and $\omega(1-\sigma_R)$ for penicillin-resistant strains, where $\omega$ represents the rate of antibiotic action, and $\sigma_S$ (respectively $\sigma_R$) the probability of non-decolonization of penicillin-susceptible (resp. penicillin-resistant) under antibiotic treatment. The two parameters $\sigma_S$ and $\sigma_R$ were first fixed for each antibiotic class, as follows. Penicillins are highly effective at clearing penicillin-susceptible pneumococci, and also partly effective at clearing penicillin-resistant pneumococci[14,15]. Therefore, we assumed that penicillin use cleared all susceptible pneumococci (that is, $\sigma_S^P = 0$) and 50% of resistant pneumococci ($\sigma_R^P = 0.5$). In contrast, cephalosporins and macrolides are less effective at clearing penicillin-susceptible pneumococci, and have little effect, if any, on penicillin-resistant

pneumococci[14,15]; therefore, we fixed $\sigma_S^C = 0.5$ and $\sigma_R^C = 1$ for cephalosporin use, and $\sigma_S^M = 0.25$ and $\sigma_R^M = 1$ for macrolides.

Second, we assumed that, under antibiotics, noncarriers had a lower risk of acquiring a susceptible strain (relative risk $\phi_S<1$) and a higher risk of acquiring a resistant strain (relative risk $\phi_R>1$). Again, these two parameters were fixed for each antibiotic class. Penicillins are narrow-spectrum antibiotics that barely affect the nasopharyngeal commensal flora; it has been argued that penicillins do not increase the relative risk of acquisition of resistant strains[16]. Therefore, we fixed $\phi_S^P = 0.1$ and $\phi_R^P = 1$ for penicillin use. Cephalosporins and macrolides, however, impact the resistant ecology more markedly[17]; therefore, for these two classes, we fixed[18]: $\phi_S^{C,M} = 0.3$ and $\phi_R^{C,M} = 4$.

The final values (parameters without superscripts) were calculated as an average of the class-specific parameters, weighted by the relative frequency of the different antibiotic classes. The fixed values for each antibiotic class and the final values used in the model are summarized in Table S3. Despite uncertainty in these parameters, we show that our results were robust to their exact values (SI, Text S3).

**Observation model.** To account for the biennial pattern of NRCP notification, we defined two probabilities, which were fixed by comparison with data from the French Medicalization Program of Information System (MPIS, Supplementary Text S2). This gave average notification probabilities of 65% for odd years, and 53% for even years. We then applied a Poisson law to relate the observed number of PM cases to the true number PM cases.

**Model equations.** We adopted the following notations. The population was divided into noncarriers *S* and carriers *C*, who were subdivided into four different categories, depending on vaccination status and antibiotic-exposure status: unexposed and unvaccinated (superscript UNV),

exposed and unvaccinated (superscript ENV), unexposed and vaccinated (superscript UV), and exposed and vaccinated (superscript EV). For each of the four classes, carriers were further subdivided into 4 categories, representing the strain carried: vaccine-serotype, penicillin-susceptible strain (subscript VS); vaccine-serotype, penicillin-resistant strain (subscript VR); nonvaccine-serotype, penicillin-susceptible strain (subscript NVS); and nonvaccine-serotype, penicillin-resistant strain (subscript NVR). To compare the model outputs with the data, the numbers of individuals infected ($I$) by each type—$I_{VS}$, $I_{VR}$, $I_{NVS}$, $I_{NVR}$—were added up for each month and then reset at the beginning of the month. Because pneumococcal meningitis is a rare infection, we assumed, for simplicity, that infection events did not deplete the pool of carriers. This formulation led to 24 state variables—4 $S$ compartments, 16 $C$ compartments, and 4 $I$ compartments.

The total numbers of carriers for each type were:

$$
\begin{aligned}
TC_{VS} &= C_{VS}^{UNV} + C_{VS}^{ENV} + C_{VS}^{UV} + C_{VS}^{EV} \\
TC_{VR} &= C_{VR}^{UNV} + C_{VR}^{ENV} + C_{VR}^{UV} + C_{VR}^{EV} \\
TC_{NVS} &= C_{NVS}^{UNV} + C_{NVS}^{ENV} + C_{NVS}^{UV} + C_{NVS}^{EV} \\
TC_{NVR} &= C_{NVR}^{UNV} + C_{NVR}^{ENV} + C_{NVR}^{UV} + C_{NVR}^{EV}
\end{aligned}
$$

The strain-specific forces of colonization, $\lambda_X$, were given by:

$$
\begin{aligned}
\lambda_{VS} &= \beta_{VS} TC_{VS} / N \\
\lambda_{VR} &= \beta_{VR} TC_{VR} / N \\
\lambda_{NVS} &= \beta_{NVS} TC_{NVS} / N \\
\lambda_{NVR} &= \beta_{NVR} TC_{NVR} / N
\end{aligned}
$$

where $N$ is the population size, and $\beta_X$ the strain-specific transmission rates, assumed to be constant over time. The strain-specific infection rates were modeled as:

$$\rho_{X,t} = \rho_X(1 + \hat{\rho}\cos(2\pi(t + \phi_\rho)))$$

where $\rho_X$ is the mean infection rate, $\hat{\rho}$ the amplitude of seasonality, *t* the time (in years) and $\phi_\rho$ the phase, which controls the time-location peak. This formulation led to the following deterministic differential equations; in the simulations, we used a stochastic analogue of these equations with Gillespie's τ-leap algorithm. Table S4 presents the complete list of model parameters.

**Equations in the unexposed and unvaccinated population (superscript UNV)**

$$\begin{aligned}
\frac{dS^{UNV}}{dt} &= (1-v(t))\mu N - (\lambda_{VS}+\lambda_{VR}+\lambda_{NVS}+\lambda_{NVR})S^{UNV} \\
&\quad +\gamma_{VS}C_{VS}^{UNV}+\gamma_{VR}C_{VR}^{UNV}+\gamma_{NVS}C_{NVS}^{UNV}+\gamma_{NVR}C_{NVR}^{UNV} \\
&\quad -\tau(t)S^{UNV}+\nu S^{ENV}-mS^{UNV} \\
\frac{dC_{VS}^{UNV}}{dt} &= \lambda_{VS}S^{UNV}-\gamma_{VS}C_{VS}^{UNV}+\theta\lambda_{VS}(C_{VR}^{UNV}+C_{NVS}^{UNV}+C_{NVR}^{UNV}) \\
&\quad -\theta(\lambda_{VR}+\lambda_{NVS}+\lambda_{NVR})C_{VS}^{UNV} \\
&\quad -\tau(t)C_{VS}^{UNV}+\nu C_{VS}^{ENV}-mC_{VS}^{UNV} \\
\frac{dC_{VR}^{UNV}}{dt} &= \lambda_{VR}S^{UNV}-\gamma_{VR}C_{VR}^{UNV}+\theta\lambda_{VR}(C_{VS}^{UNV}+C_{NVS}^{UNV}+C_{NVR}^{UNV}) \\
&\quad -\theta(\lambda_{VS}+\lambda_{NVS}+\lambda_{NVR})C_{VR}^{UNV} \\
&\quad -\tau(t)C_{VR}^{UNV}+\nu C_{VR}^{ENV}-mC_{VR}^{UNV} \\
\frac{dC_{NVS}^{UNV}}{dt} &= \lambda_{NVS}S^{UNV}-\gamma_{NVS}C_{NVS}^{UNV}+\theta\lambda_{NVS}(C_{VS}^{UNV}+C_{VR}^{UNV}+C_{NVR}^{UNV}) \\
&\quad -\theta(\lambda_{VS}+\lambda_{VR}+\lambda_{NVR})C_{NVS}^{UNV} \\
&\quad -\tau(t)C_{NVS}^{UNV}+\nu C_{NVS}^{ENV}-mC_{NVS}^{UNV} \\
\frac{dC_{NVR}^{UNV}}{dt} &= \lambda_{NVR}S^{UNV}-\gamma_{NVR}C_{NVR}^{UNV}+\theta\lambda_{NVR}(C_{VS}^{UNV}+C_{VR}^{UNV}+C_{NVS}^{UNV}) \\
&\quad -\theta(\lambda_{VS}+\lambda_{VR}+\lambda_{NVS})C_{NVR}^{UNV} \\
&\quad -\tau(t)C_{NVR}^{UNV}+\nu C_{NVR}^{ENV}-mC_{NVR}^{UNV}
\end{aligned}$$

**Equations in the exposed and unvaccinated population (superscript ENV)**

$$\begin{aligned}
\frac{dS^{ENV}}{dt} &= -[\phi_S(\lambda_{VS}+\lambda_{NVS})+\phi_R(\lambda_{VR}+\lambda_{NVR})]S^{ENV} \\
&\quad +[\gamma_{VS}+\omega(1-\sigma_S)]C_{VS}^{ENV}+[\gamma_{NVS}+\omega(1-\sigma_S)]C_{NVS}^{ENV} \\
&\quad +[\gamma_{VR}+\omega(1-\sigma_R)]C_{VR}^{ENV}+[\gamma_{NVR}+\omega(1-\sigma_R)]C_{NVR}^{ENV} \\
&\quad +\tau(t)S^{UNV}-\nu S^{ENV}-mS^{ENV} \\
\frac{dC_{VS}^{ENV}}{dt} &= \phi_S\lambda_{VS}[S^{ENV}+\theta(C_{VR}^{ENV}+C_{NVS}^{ENV}+C_{NVR}^{ENV})] \\
&\quad -[\gamma_{VS}+\omega(1-\sigma_S)]C_{VS}^{ENV}-\theta[\phi_R(\lambda_{VR}+\lambda_{NVR})+\phi_S\lambda_{NVS})]C_{VS}^{ENV} \\
&\quad +\tau(t)C_{VS}^{UNV}-\nu C_{VS}^{ENV}-mC_{VS}^{ENV} \\
\frac{dC_{VR}^{ENV}}{dt} &= \phi_R\lambda_{VR}[S^{ENV}+\theta(C_{VS}^{ENV}+C_{NVS}^{ENV}+C_{NVR}^{ENV})] \\
&\quad -[\gamma_{VR}+\omega(1-\sigma_R)]C_{VR}^{ENV}-\theta[\phi_S(\lambda_{VS}+\lambda_{NVS})+\phi_R\lambda_{NVR}]C_{VR}^{ENV} \\
&\quad +\tau(t)C_{VR}^{UNV}-\nu C_{VR}^{ENV}-mC_{VR}^{ENV} \\
\frac{dC_{NVS}^{ENV}}{dt} &= \phi_S\lambda_{NVS}[S^{ENV}+\theta(C_{VS}^{ENV}+C_{VR}^{ENV}+C_{NVR}^{ENV})] \\
&\quad -[\gamma_{NVS}+\omega(1-\sigma_S)]C_{NVS}^{ENV}-\theta[\phi_S\lambda_{VS}+\phi_R(\lambda_{VR}+\lambda_{NVR})]C_{NVS}^{ENV} \\
&\quad +\tau(t)C_{NVS}^{UNV}-\nu C_{NVS}^{ENV}-mC_{NVS}^{ENV} \\
\frac{dC_{NVR}^{ENV}}{dt} &= \phi_R\lambda_{NVR}[S^{ENV}+\theta(C_{VS}^{ENV}+C_{VR}^{ENV}+C_{NVS}^{ENV})] \\
&\quad -[\gamma_{NVR}+\omega(1-\sigma_R)]C_{NVR}^{ENV}-\theta[\phi_S(\lambda_{VS}+\lambda_{NVS})+\phi_R\lambda_{VR}]C_{NVR}^{ENV} \\
&\quad +\tau(t)C_{NVR}^{UNV}-\nu C_{NVR}^{ENV}-mC_{NVR}^{ENV}
\end{aligned}$$

**Equations in the unexposed and vaccinated population (superscript UV)**

$$
\begin{aligned}
\frac{dS^{UV}}{dt} &= v(t)\mu N - [(1-\sigma_V)(\lambda_{VS}+\lambda_{VR})+\lambda_{NVS}+\lambda_{NVR}]S^{UV} \\
&\quad +\gamma_{VS}C^{UV}_{VS}+\gamma_{VR}C^{UV}_{VR}+\gamma_{NVS}C^{UV}_{NVS}+\gamma_{NVR}C^{UV}_{NVR} \\
&\quad -\tau(t)S^{UV}+\nu S^{EV}-mS^{UV} \\
\frac{dC^{UV}_{VS}}{dt} &= (1-\sigma_V)\lambda_{VS}S^{UV}-\gamma_{VS}C^{UV}_{VS}+\theta(1-\sigma_V)\lambda_{VS}(C^{UV}_{VR}+C^{UV}_{NVS}+C^{UV}_{NVR}) \\
&\quad -\theta[(1-\sigma_V)\lambda_{VR}+\lambda_{NVS}+\lambda_{NVR}]C^{UV}_{VS} \\
&\quad -\tau(t)C^{UV}_{VS}+\nu C^{EV}_{VS}-mC^{UV}_{VS} \\
\frac{dC^{UV}_{VR}}{dt} &= (1-\sigma_V)\lambda_{VR}S^{UV}-\gamma_{VR}C^{UV}_{VR}+\theta(1-\sigma_V)\lambda_{VR}(C^{UV}_{VS}+C^{UV}_{NVS}+C^{UV}_{NVR}) \\
&\quad -\theta[(1-\sigma_V)\lambda_{VS}+\lambda_{NVS}+\lambda_{NVR}]C^{UV}_{VR} \\
&\quad -\tau(t)C^{UV}_{VR}+\nu C^{EV}_{VR}-mC^{UV}_{VR} \\
\frac{dC^{UV}_{NVS}}{dt} &= \lambda_{NVS}S^{UV}-\gamma_{NVS}C^{UV}_{NVS}+\theta\lambda_{NVS}(C^{UV}_{VS}+C^{UV}_{VR}+C^{UV}_{NVR}) \\
&\quad -\theta[(1-\sigma_V)(\lambda_{VS}+\lambda_{VR})+\lambda_{NVR}]C^{UV}_{NVS} \\
&\quad -\tau(t)C^{UV}_{NVS}+\nu C^{EV}_{NVS}-mC^{UV}_{NVS} \\
\frac{dC^{UV}_{NVR}}{dt} &= \lambda_{NVR}S^{UV}-\gamma_{NVR}C^{UV}_{NVR}+\theta\lambda_{NVR}(C^{UV}_{VS}+C^{UV}_{VR}+C^{UV}_{NVS}) \\
&\quad -\theta[(1-\sigma_V)(\lambda_{VS}+\lambda_{VR})+\lambda_{NVS}]C^{UV}_{NVR} \\
&\quad -\tau(t)C^{UV}_{NVR}+\nu C^{EV}_{NVR}-mC^{UV}_{NVR}
\end{aligned}
$$

**Equations in the exposed and vaccinated population (superscript EV)**

$$
\begin{aligned}
\frac{dS^{EV}}{dt} &= -[(1-\sigma_V)(\phi_S\lambda_{VS}+\phi_R\lambda_{VR})+\phi_S\lambda_{NVS}+\phi_R\lambda_{NVR}]S^{EV} \\
&\quad +[\gamma_{VS}+\omega(1-\sigma_S)]C_{VS}^{EV}+[\gamma_{VR}+\omega(1-\sigma_R)]C_{VR}^{EV} \\
&\quad +[\gamma_{NVS}+\omega(1-\sigma_S)]C_{NVS}^{EV}+[\gamma_{NVR}+\omega(1-\sigma_R)]C_{NVR}^{EV} \\
&\quad +\tau(t)S^{UV}-\nu S^{EV}-mS^{EV} \\
\frac{dC_{VS}^{EV}}{dt} &= (1-\sigma_V)\phi_S\lambda_{VS}[S^{EV}+\theta(C_{VR}^{EV}+C_{NVS}^{EV}+C_{NVR}^{EV})] \\
&\quad -[\gamma_{VS}+\omega(1-\sigma_S)]C_{VS}^{EV}-\theta[(1-\sigma_V)\phi_R\lambda_{VR}+\phi_S\lambda_{NVS}+\phi_R\lambda_{NVR}]C_{VS}^{EV} \\
&\quad +\tau(t)C_{VS}^{UV}-\nu C_{VS}^{EV}-mC_{VS}^{EV} \\
\frac{dC_{VR}^{EV}}{dt} &= (1-\sigma_V)\phi_R\lambda_{VR}[S^{EV}+\theta(C_{VS}^{EV}+C_{NVS}^{EV}+C_{NVR}^{EV})] \\
&\quad -[\gamma_{VR}+\omega(1-\sigma_R)]C_{VR}^{EV}-\theta[(1-\sigma_V)\phi_S\lambda_{VS}+\phi_S\lambda_{NVS}+\phi_R\lambda_{NVR}]C_{VR}^{EV} \\
&\quad +\tau(t)C_{VR}^{UV}-\nu C_{VR}^{EV}-mC_{VR}^{EV} \\
\frac{dC_{NVS}^{EV}}{dt} &= \phi_S\lambda_{NVS}[S^{EV}+\theta(C_{VS}^{EV}+C_{VR}^{EV}+C_{NVR}^{EV})] \\
&\quad -[\gamma_{NVS}+\omega(1-\sigma_S)]C_{NVS}^{EV}-\theta[(1-\sigma_V)(\phi_S\lambda_{VS}+\phi_R\lambda_{VR})+\phi_R\lambda_{NVR}]C_{NVS}^{EV} \\
&\quad +\tau(t)C_{NVS}^{UV}-\nu C_{NVS}^{EV}-mC_{NVS}^{EV} \\
\frac{dC_{NVR}^{EV}}{dt} &= \phi_R\lambda_{NVR}[S^{EV}+\theta(C_{VS}^{EV}+C_{VR}^{EV}+C_{NVS}^{EV})] \\
&\quad -[\gamma_{NVR}+\omega(1-\sigma_R)]C_{NVR}^{EV}-\theta[(1-\sigma_V)(\phi_S\lambda_{VS}+\phi_R\lambda_{VR})+\phi_S\lambda_{NVS}]C_{NVR}^{EV} \\
&\quad +\tau(t)C_{NVR}^{UV}-\nu C_{NVR}^{EV}-mC_{NVR}^{EV}
\end{aligned}
$$

**Equations for the number infected**

$$
\begin{aligned}
\frac{dI_{VS}}{dt} &= \rho_{VS}(t)\times(C_{VS}^{UNV}+C_{VS}^{ENV}) \\
\frac{dI_{VR}}{dt} &= \rho_{VR}(t)\times(C_{VR}^{UNV}+C_{VR}^{ENV}) \\
\frac{dI_{NVS}}{dt} &= \rho_{NVS}(t)\times TC_{NVS} \\
\frac{dI_{NVR}}{dt} &= \rho_{NVR}(t)\times TC_{NVR}
\end{aligned}
$$

**Typical model outputs.** In Fig. S3, we present typical model outputs for the population size, the number of vaccinees, and the number of individuals under antibiotic treatment. Because these outputs varied little across simulations, results of only one simulation are given.

**Derivation of the VNV and SR models.** As explained in the Main Text, we formulated two hypotheses regarding the cause of serotype replacement in PM, which were translated in two distinct models.

The Vaccine/NonVaccine (VNV) model integrated possible transmissibility and/or invasiveness differences between vaccine and nonvaccine serotypes. Therefore, for this model:

1. $\beta_V = \beta_{VS} = \beta_{VR}$ and $\beta_{NV} = \beta_{NVS} = \beta_{NVR}$ (equalities of transmission rates)
2. $\rho_V = \rho_{VS} = \rho_{VR}$ and $\rho_{NV} = \rho_{NVS} = \rho_{NVR}$ (equalities of PM rates)

In practice, we estimated ($\beta_V$, $\beta_{NV}/\beta_V$) and ($\rho_V$, $\rho_{NV}/\rho_V$) for this model.

The Susceptible/Resistant (SR) model integrated possible transmissibility and/or invasiveness differences between penicillin-susceptible and penicillin-resistant pneumococci. Therefore, for this model:

1. $\beta_S = \beta_{VS} = \beta_{NVS}$ and $\beta_R = \beta_{VR} = \beta_{NVR}$ (equalities of transmission rates)
2. $\rho_S = \rho_{VS} = \rho_{NVS}$ and $\rho_R = \rho_{VR} = \rho_{NVR}$ (equalities of PM rates)

Similarly, we estimated ($\beta_S$, $\beta_R/\beta_S$) and ($\rho_S$, $\rho_R/\rho_S$) for this model.

**Parameter estimation and model comparison.** The data consisted of monthly PM-incidence data for each strain, i.e., parallel time-series $I_{VS,t}^o$, $I_{VR,t}^o$,$I_{NVS,t}^o$, and $I_{NVR,t}^o$, where the superscript o refers to observed data. For each strain, these data were related to the true number of PM cases via a Poisson distribution. Specifically, denoting $I_{VS,t}$ the true number of VS-strain PM and $\phi(t)$ the notification probability to NRCP ($\phi(t) = \phi$ for odd years or $\phi(t) = \psi$ for even years), the observation model was $I_{VS,t}^o \sim \text{Poisson}(\phi(t)I_{VS,t})$. Therefore, the likelihood was the product of four components, relating the true number of PM cases to the data for each strain.

For the parameters $\beta_S$ and $\beta_V$, i.e., absolute transmission rates, preliminary analyses showed a nonidentifiability, which reflected the lack of information in the data on absolute

carriage levels in the population. To address this, we added a fifth component in the likelihood, $L_C \approx N(c_0,0.1^2)$, where $c_0$ is the initial carriage prevalence (Table S4). This distribution was chosen to add a baseline value for carriage prevalence in the population, but with a sufficiently large variance to allow variations over time, e.g., related to vaccination and reduced antibiotic use. In practice, this solved the problem of identifiability for the two parameters by pushing their values to regions that resulted in carriage prevalence close to $c_0$. For completeness, we also performed estimations without adding $L_C$; the other parameter estimates were identical (data not shown).

The likelihood was maximized with respect to the parameters using the maximum iterated filtering (MIF) algorithm[19]. This estimation procedure requires the specification of algorithmic parameters, which, once convergence has been reached, play no role in the results. For all estimations, we used the same initial variance multiplier, $c^2$=9, and the same cooling factor, $\alpha$=0.95. Maximum likelihood estimates were computed as the mean of 10 MIF replicates with 10,000 particles and 150 filtering operations. For likelihood-profile computations, we fixed each parameter at different values in a prespecified range and computed maximum likelihood estimates for the other parameters, as the mean of two MIF replicates with 2,000 particles and 60 filtering operations. The profile was then smoothed using a local quadratic regression (implemented in the R loess function, span of 0.75), and the 99% univariate confidence interval was taken to be $\chi_1^2(0.99)/2\approx3.32$ units below the maximum.

**Text S2. Supplementary data**

**Pneumococcal meningitis (PM) trends by age class (Fig. S1)**

**Comparison of National Reference Center for Pneumococci (NRCP) versus Medicalization Program of Information System (MPIS) data.** To assess the exhaustiveness of the NRCP data, we compared annual PM numbers notified to NRCP with those reported to the MPIS (Table S1). This comparison did not reveal any trend in the notification to NRCP; however, notification differed systematically between odd and even years. Some Regional Pneumococcus Observatories do not send their pneumococcal isolates to NRCP on even years, thereby explaining this pattern. Therefore, we fixed two notification probabilities from Table S1:

1. $\phi = \frac{395+430+430+482}{603+634+643+781} = 0.65$

2. $\psi = \frac{324+325+318+379}{555+666+609+706} = 0.53$

**Vaccine coverage by birth cohort (Table S2).**

## Text S3. Detailed results and supplementary analyses

**Parameter estimates.** For the parameter estimates for the VNV and SR models, see Table S5 and Fig. S4. The transmissibility and invasiveness-ratio values are discussed in the Main Text.

Here, we discuss the values of the other parameters. In the SR model, the estimated value of the competition parameter, $\theta$, indicates moderate-to-null between-strain competition for acquisition (acquisition risk ratio 0.6–1). Previous studies that considered a similar competition mechanism indicated moderate[3,4] to elevated[20] levels of competition in children. A recent study analyzed longitudinal datasets of colonization among children in several countries, and provided evidence of strong competition, despite high variability of the estimates[21]. To what extent those results, obtained in children, can be compared to our estimate in a general population is not straightforward. Indeed, carriage duration decreases with age, thereby progressively limiting competition among pneumococci. Therefore, because of the lack of age-structure, the numerical value of our competition parameter may be difficult to interpret. This interpretation is further complicated by the existence of immune mechanisms responding to carriage, co-carriage in the nasopharynx, etc., which were beyond the scope of this study.

In the VNV and SR models, vaccine effectiveness against carriage was estimated to be high (>75%), a value higher than previous estimates in 50%–60% range[11], but should be interpreted with caution. Indeed, in any vaccination model, the proportion of children protected by the vaccine is given by $e*p$, where $p$ is the proportion of vaccinated children and $e$ the vaccine effectiveness[22]. Therefore, any misspecification of the vaccine coverage $p$ is compensated by a change in the estimated vaccine effectiveness $e$, which might explain the high value we obtained.

**Sensitivity analyses.** To test the robustness of our results, we conducted a series of sensitivity analyses. In particular, we sought to determine whether the misspecification of antibiotic-related parameters (see Text S1) might have impacted our main results on fitness costs; i.e., parameters

$\beta_R/\beta_S$ and $\rho_R/\rho_S$. To do so, we varied the values of several pairs of parameters and performed the estimations as before.

Fig. S5 presents the sensitivity-analysis results for $\beta_R/\beta_S$, when varying $\phi_S$ and $\phi_R$. Increasing $\phi_R$ values shifted the competitive balance in favor of resistant strains, an effect counterbalanced by higher estimated fitness costs; the opposite was true for $\phi_S$. More generally, the estimated fitness costs increased for higher values of parameters favoring resistant strains (e.g., $\sigma_R$ and $\phi_R$) and decreased for higher values of parameters favoring susceptible strains (e.g., $\sigma_S$ and $\phi_S$). The fitness costs also increased with longer durations of carriage $1/\gamma$. These findings are consistent with theory, and were reported previously[23].

Table S6 gives the results of all the analyses. For all the parameters tested, the estimates varied little (overall range of estimates: $\beta_R/\beta_S$ in 0.90–0.98 and $\rho_R/\rho_S$ in 0.64–0.90); crucially, the estimates always highlighted the existence of the fitness costs.

**Effect of serotype 19A.** As shown on Fig. S7, serotype 19A accounted for the majority of NVR PM during the study period. Because our best (SR) model imperfectly reproduced the late rise of NVR PM, we attempted to assess the individual effect of serotype 19A. To do so, we created a new dataset by removing serotype 19A-PM and performed the estimations as before in the VNV and SR models.

In the VNV model, parameter estimates changed little, except for the estimated $\rho_{NV}/\rho_V$ value (99% CI) that changed from 1.01 (0.81–1.26) to 0.88 (0.71–1.07). That change suggested lower invasiveness of nonvaccine serotypes when serotype 19A was removed; in contrast, the estimated value (99% CI) of $\beta_{NV}/\beta_V$, 1.00 (0.99–1.01), remained unchanged.

In the SR model, the estimation suggested higher fitness cost on transmissibility, $\beta_R/\beta_S$ value (99% CI) changed from 0.954 (0.950–0.957) to 0.952 (0.948–0.956), and on invasiveness, $\rho_R/\rho_S$ value (99% CI) changed from 0.78 (0.54–0.93) to 0.76 (0.51–0.92). The model fit to the

data without serotype 19A, presented in Fig. S7, was acceptable for NVR-strain PM, although the predictive power was limited because of the low number of NVR-strain PM remaining in the dataset ($R^2 = 0.27$).

These outcomes show that the dynamics of penicillin-resistant serotype 19A added to the increase of NVS PM caused by reduced antibiotic use. The distinct serotype-19A pattern was previously observed in France[24], possibly reflecting the clonal expansion of ST276[25]. These results also suggest that serotype 19A might not pay a fitness cost for resistance[26].

## Supplementary Figures

**Fig. S1. 2001–2009 PM incidence in individuals [0, 3) years old (top panel), [3, 16) years old (middle panel), and ≥16 years old (bottom panel).** The *y*-axis differs in the three panels. VS: vaccine-serotype penicillin-susceptible meningitis; VR: vaccine-serotype penicillin-resistant meningitis; NVS: nonvaccine-serotype penicillin-susceptible meningitis; NVR: nonvaccine-serotype penicillin-resistant meningitis. Black lines: yearly total PM cases.

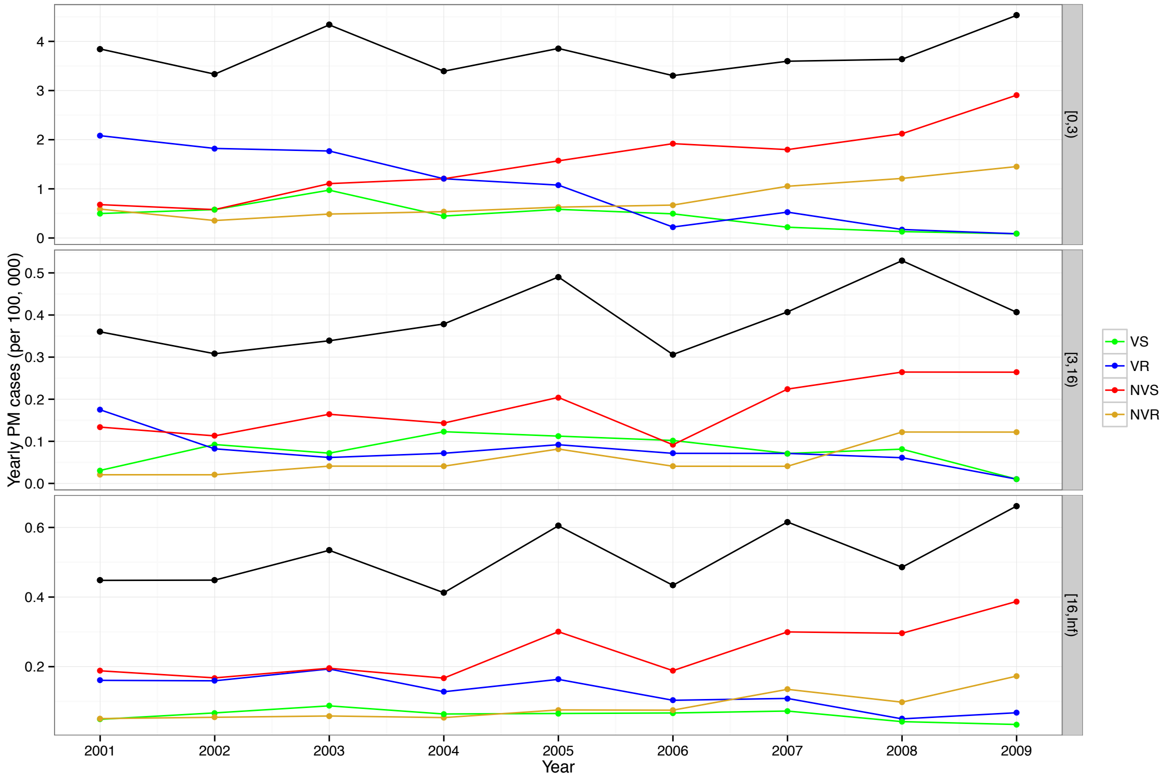

**Fig. S2. Relative frequencies of use of the three antibiotic classes over 2001–2007.** Data taken from [12].

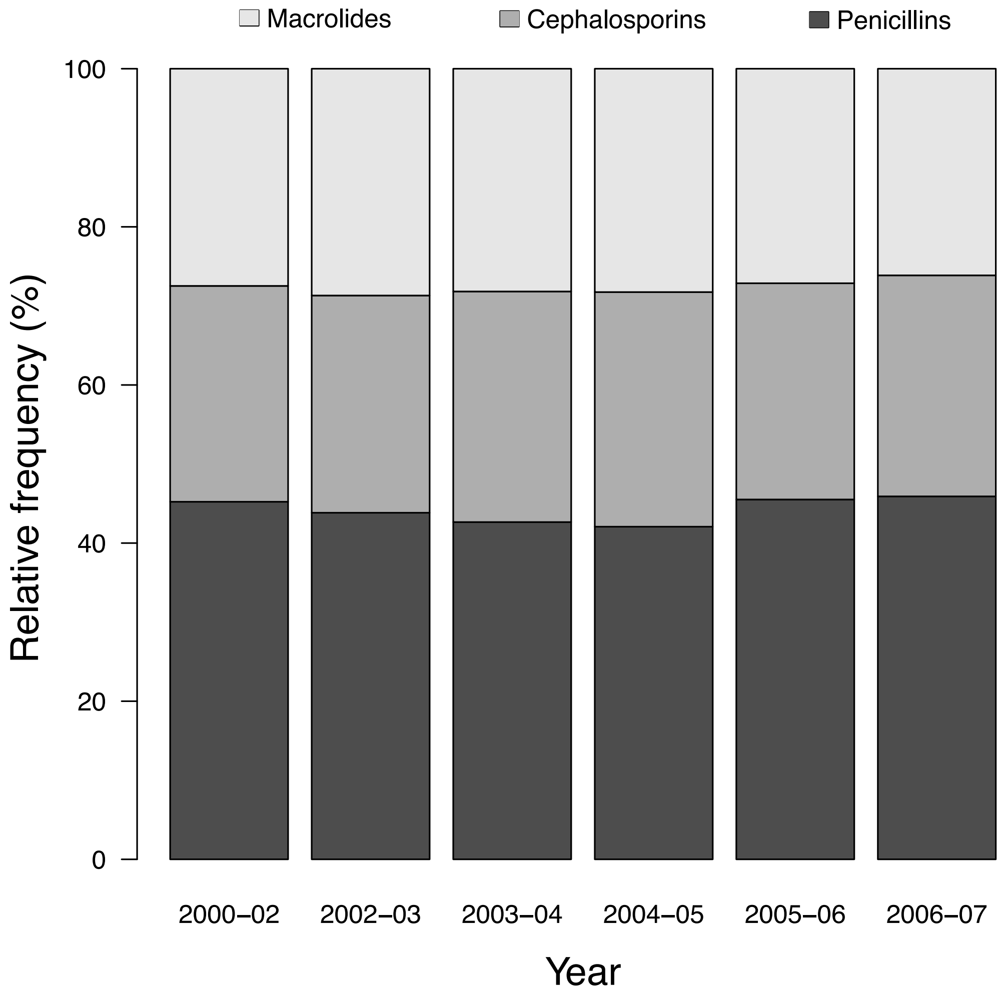

**Fig. S3. Typical model outputs.** Top: population size; Middle: number of vaccinated individuals; Bottom: number of individuals taking antibiotics.

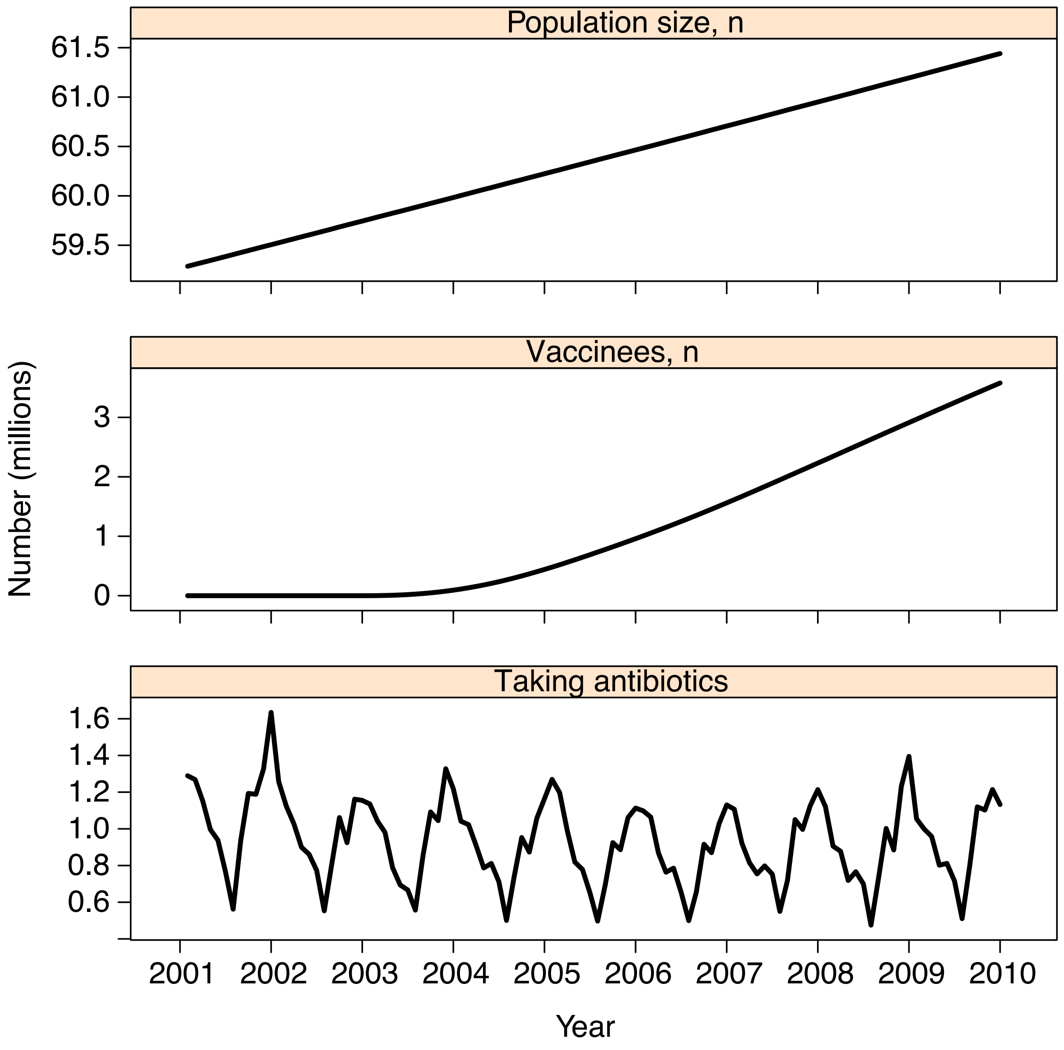

## Fig S4. Parameters profiles for the SR model.

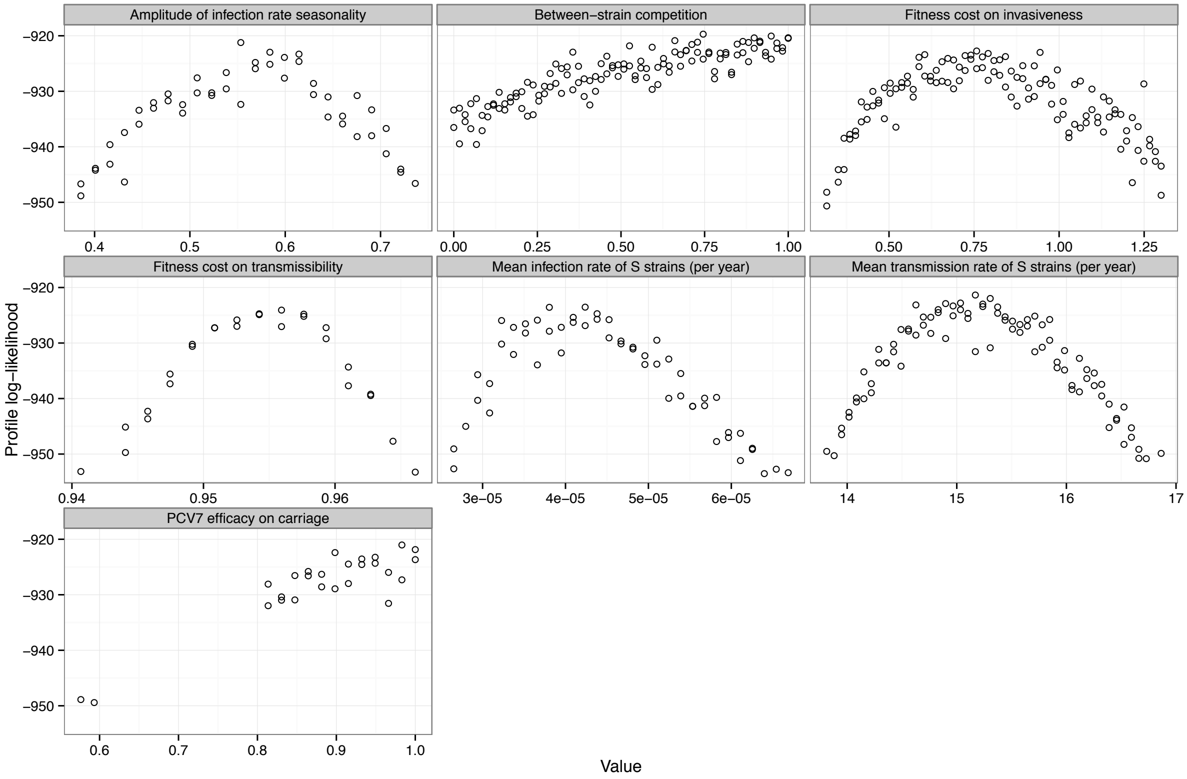

**Fig. S5. Sensitivity analysis for $\beta_R/\beta_S$ when varying $\phi_S$ and $\phi_R$.**

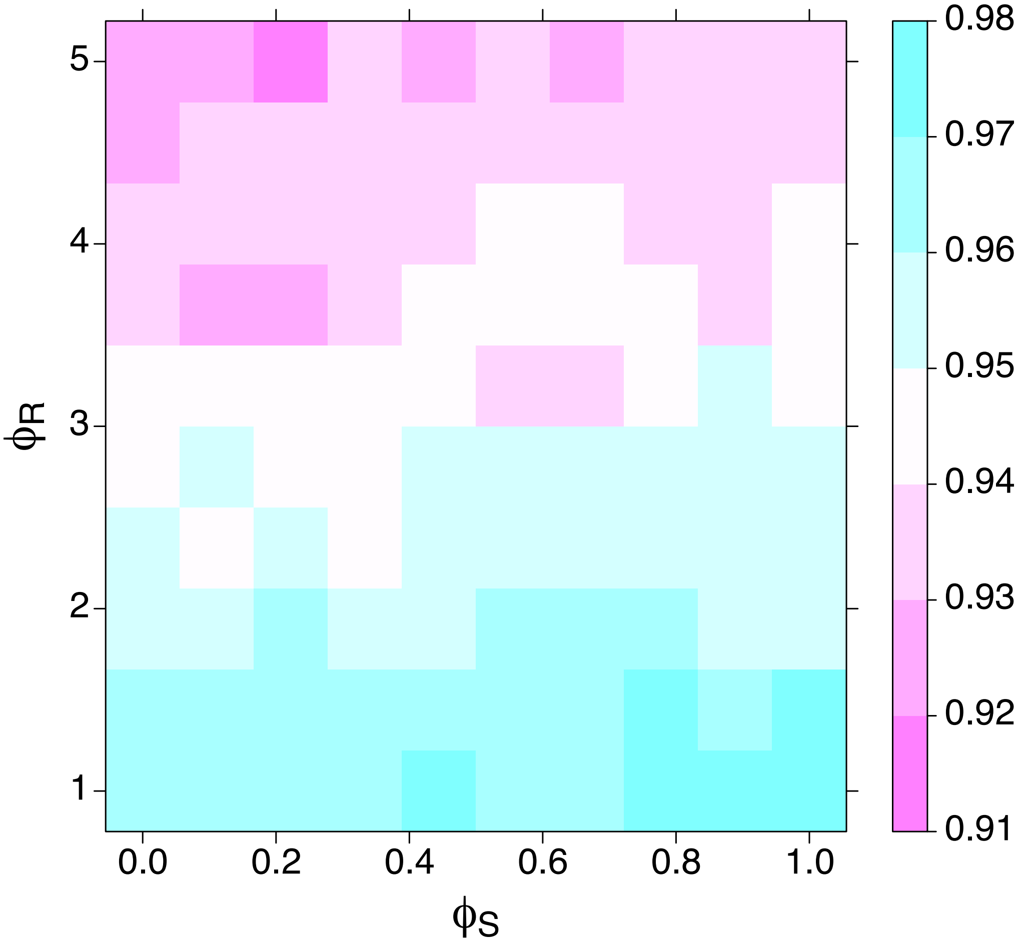

**Fig. S6. Predicted dynamics of carriage in the SR model.**

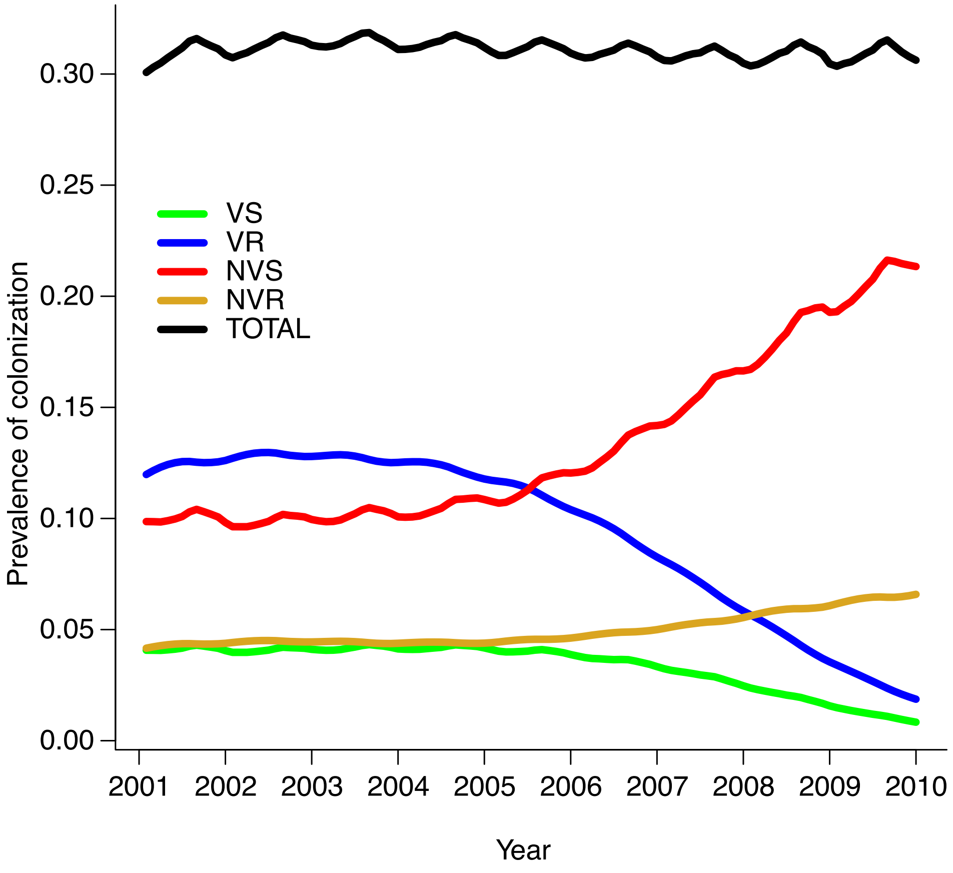

**Fig. S7. Contribution of serotype 19A (in red) to the number of NVR-PM (in black).**

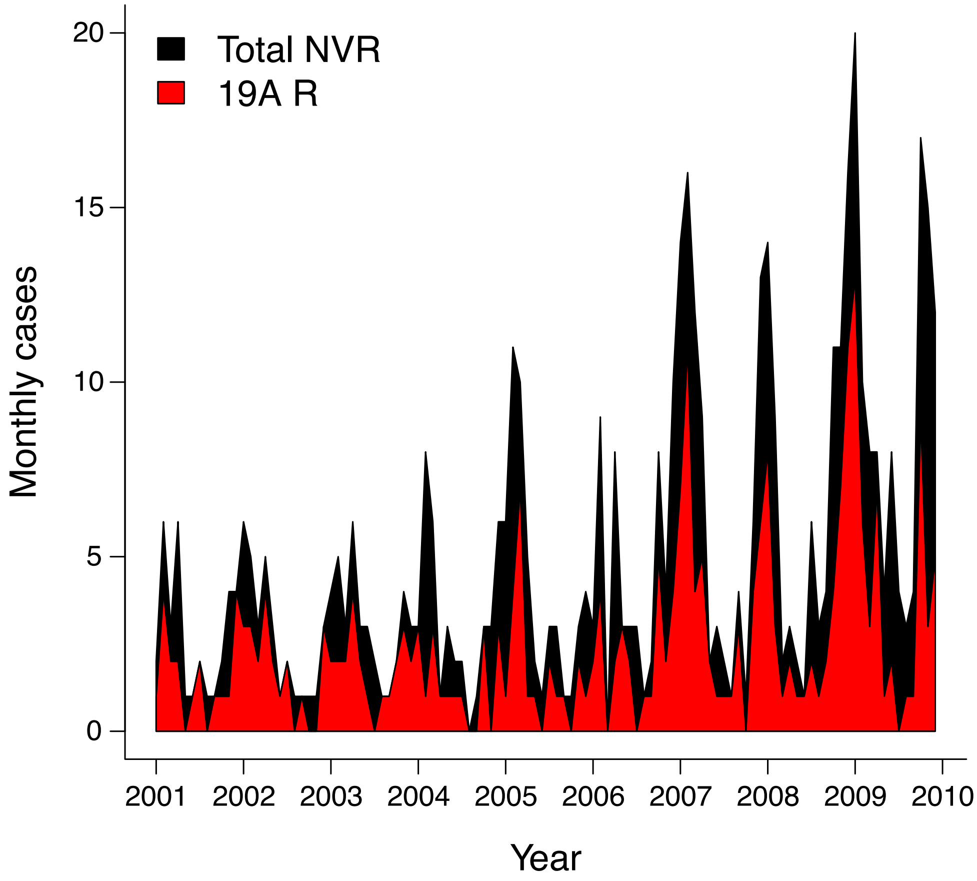

**Fig. S8. Model fit to data after removing serotype 19A.** For each strain, the observed numbers of PM (red curve) and the mean of 1,000 stochastic runs of the SR model (black curve) with their 95% prediction intervals (gray) are shown. For completeness, the total number of PM (panel TOTAL) is also given; this series was not used for statistical inference. Note the different *y*-axis for each graph. VS: vaccine-serotype penicillin-susceptible; VR: vaccine-serotype, penicillin-resistant; NVS: nonvaccine-serotype penicillin-susceptible; NVR: nonvaccine-serotype penicillin-resistant.

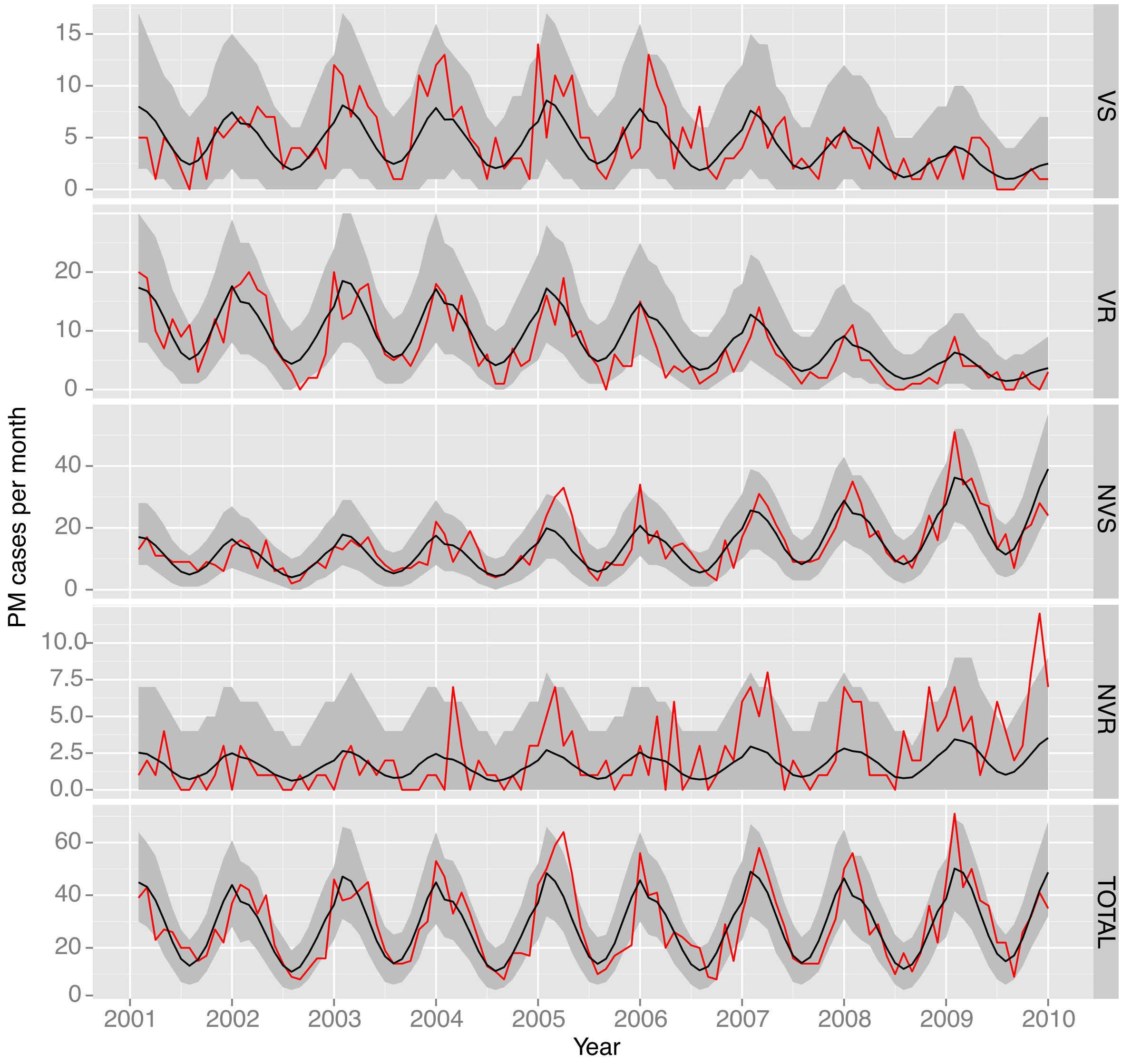

**Fig. S9. Qualitative comparison of yearly age-stratified incidences between the data (top graph) and simulations from an age-structured model (bottom graph).** We extended our model to include the following age structure: [0,3) y, [3, 16) y, and ≥16 y. Durations of carriage were assumed to decrease with age: 2 months in [0,3) y, 1 month in [3, 16) y, and 2 weeks in ≥16 y[27]. The contact matrix, $C_{ij}$, was calculated using reported daily contacts in Great Britain[28], converted to units of contacts per year. We then defined the transmission matrix, $\beta_{ij}=q_i \times C_{ij}$, where $q_i=(1.7\times10^{-2}, 4.3\times10^{-3}, 4.3\times10^{-3})$ represents age-specific probabilities of pneumococcal acquisition given exposure, calibrated to reach 50% carriage prevalence in [0, 3) y, 30% in [3, 16) y, and 10% in ≥16 y[27]. Although we did not investigate further, decreasing susceptibility with age might be explained by acquired immunity or differences in the nature of contacts (e.g., riskier contacts for younger children). Consistent with our goal to allow a qualitative comparison with the French data, we did not attempt to estimate the other age-specific parameters. In particular, age-specific mean infection rates were fixed at arbitrary values, and, for each age group, we represent the yearly incidence (per 100, 000) divided by the total incidence (per 100, 000) in 2001. Therefore, we insist that this comparison is qualitative, not quantitative. For the deterministic simulation, $\beta_R/\beta_S$ (fitness cost on transmission) was fixed at 0.945, all other parameters were fixed to values estimated (Table S5) or fixed (Table S4) in the simple model. VS: vaccine-serotype penicillin-susceptible; VR: vaccine-serotype, penicillin-resistant; NVS: nonvaccine-serotype penicillin-susceptible; NVR: nonvaccine-serotype penicillin-resistant. Note that the top graph corresponds to Fig. S1, scaled by the age-specific total incidence in 2001.

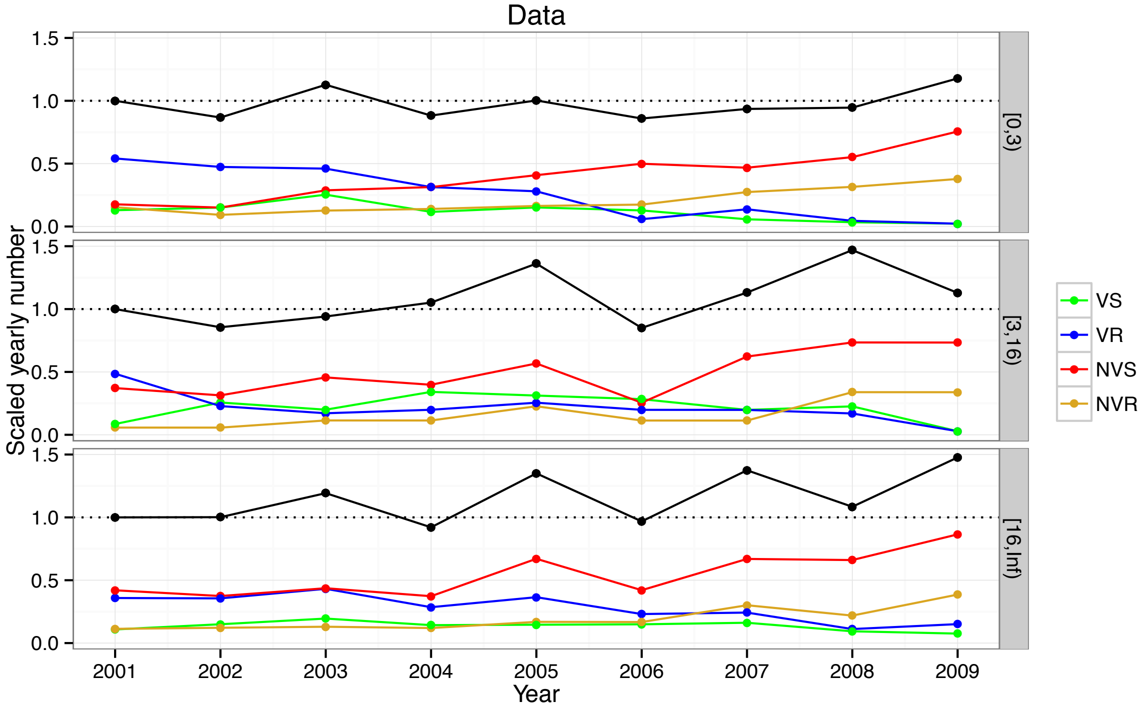

Data
Scaled yearly number
Year
2001
2002
2003
2004
2005
2006
2007
2008
2009
[0,3)
[3,16)
[16,Inf)
VS
VR
NVS
NVR


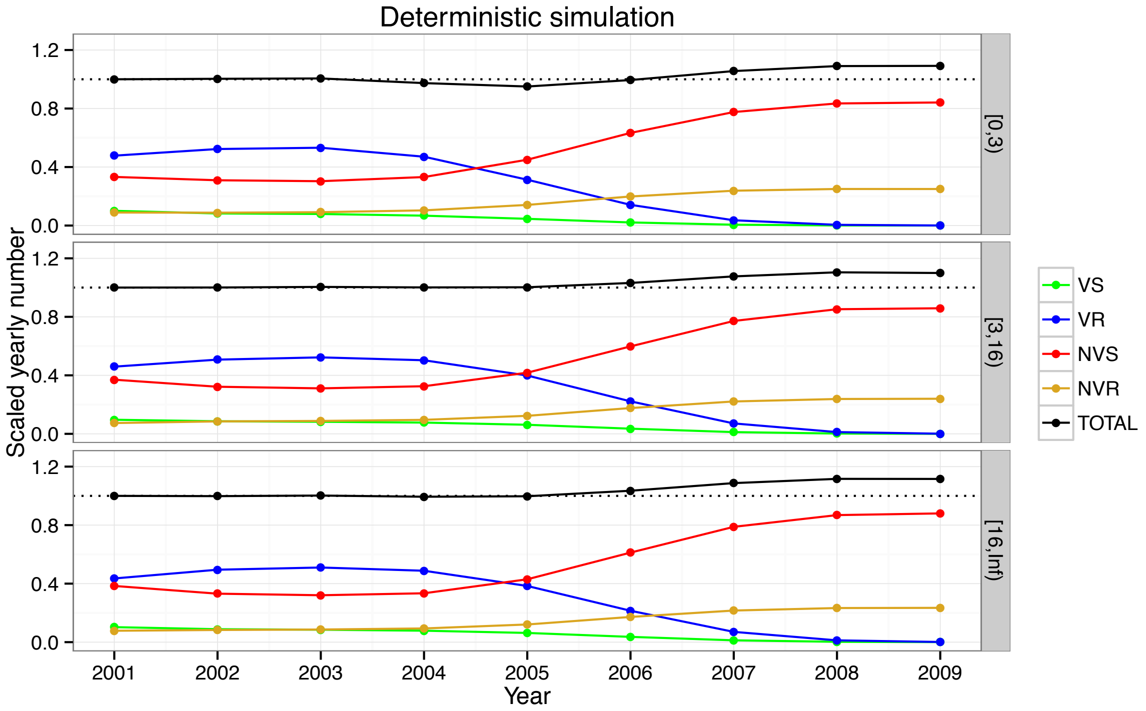

Deterministic simulation
Scaled yearly number
Year
2001
2002
2003
2004
2005
2006
2007
2008
2009
[0,3)
[3,16)
[16,Inf)
VS
VR
NVS
NVR
TOTAL

**Fig. S10. Out-of-fit predictions in 2008–2009.** We fitted the SR model to 2001–2007 data (training period) and used the resulting estimates to predict 2008–2009 data (testing period). For each strain, the observed numbers of pneumococcal meningitis (red lines) and the results of 10 stochastic runs during the training period (light grey lines) and the testing period (dark grey lines) are represented. For completeness, the total number of pneumococcal meningitis (panel TOTAL) is also given; note that this series was not used for statistical inference. R-squared values were: VS, 0.25; VR, 0.52; NVS, 0.63; NVR, –0.19; TOTAL, 0.67. Note the different *y*-axis for each graph. VS: vaccine-serotype penicillin-susceptible; VR: vaccine-serotype, penicillin-resistant; NVS: nonvaccine-serotype penicillin-susceptible; NVR: nonvaccine-serotype penicillin-resistant.

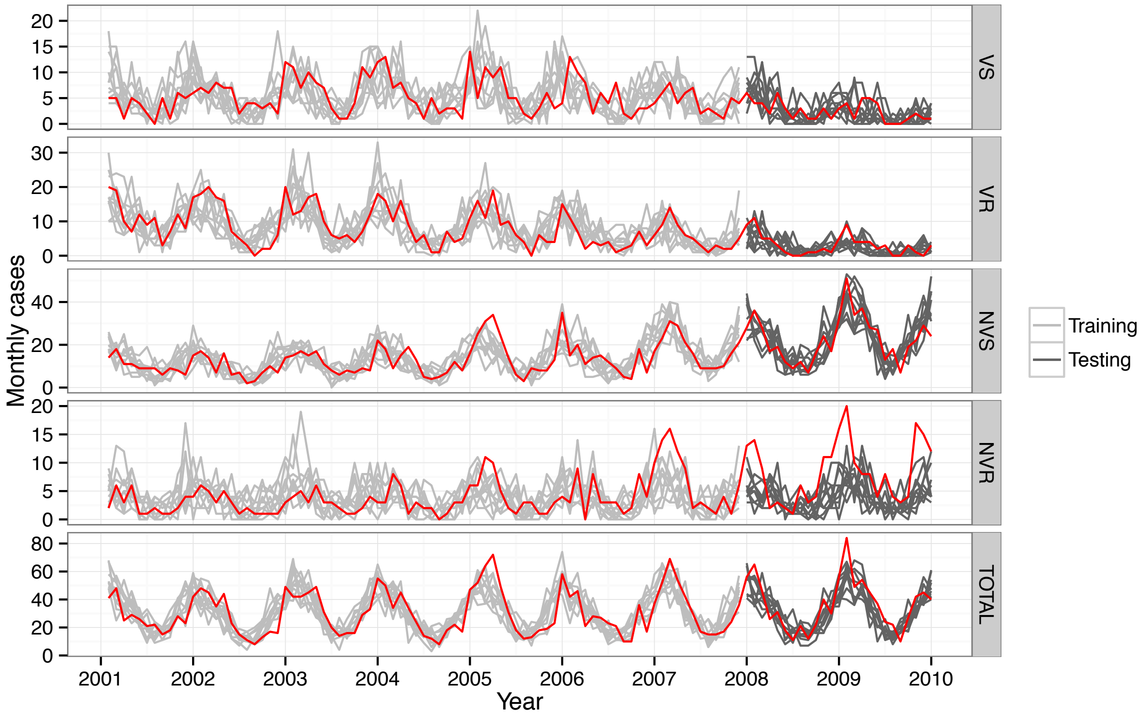

**Supplementary Tables**

**Table S1. Comparison of numbers of PM cases reported to NRCP and MPIS.**

| Year | NRCP, *n* | MPIS, *n* | NRCP/MPIS ratio |
|---|---|---|---|
| 2001 | — | — | — |
| 2002 | 324 | 555 | 0.58 |
| 2003 | 395 | 603 | 0.66 |
| 2004 | 325 | 666 | 0.49 |
| 2005 | 430 | 634 | 0.68 |
| 2006 | 318 | 609 | 0.52 |
| 2007 | 430 | 643 | 0.67 |
| 2008 | 379 | 706 | 0.54 |
| 2009 | 482 | 781 | 0.62 |

Abbreviations: NRCP, French National Reference Center for Pneumococci; MPIS, Medicalization Program of Information System.

**Table S2. Yearly PCV7 coverage estimates. For each birth cohort,** the estimates of vaccine coverage correspond to the proportion of children having received at least 2 doses by the age of 12 months.

| Birth cohort | Vaccine coverage |
|---|---|
| 2004 | 0.615 |
| 2005 | 0.743 |
| 2006 | 0.863 |
| 2007 | 0.913 |
| 2008 | 0.911 |
| 2009 | 0.881 |

**Table S3. Antibiotic-related parameters for each antibiotic class and final values used in the models.** The final values were computed as the average of each class's effect, weighted by their relative frequency.

| Antibiotic class | Relative frequency | Parameter | | | |
|---|---|---|---|---|---|
| | | $\sigma_S$ | $\sigma_R$ | $\phi_S$ | $\phi_R$ |
| Penicillins | 44% | 0 | 0.5 | 0.1 | 1 |
| Cephalosporins | 28% | 0.5 | 1 | 0.3 | 4 |
| Macrolides | 28% | 0.25 | 1 | 0.3 | 4 |
| Final value (weighted average) | — | 0.2 | 0.8 | 0.2 | 2.5 |

**Table S4. Model parameters.**

| Param. | Meaning | Value | Source |
|---|---|---|---|
| $\sigma_S, \sigma_R$ | Probability of non-decolonization of S, R strains following antibiotic treatment | 0.2, 0.8 (SR model) Estimated (VNV model) | Text S1 |
| $\phi_S, \phi_R$ | Relative acquisition rates under antibiotics | 0.2, 2.5 (SR model) Estimated (VNV model) | Text S1 |
| $1/\gamma$ | Mean duration of carriage | 37 days | [29] |
| $\omega$ | Rate of antibiotic action | 1/4 $\text{day}^{-1}$ | [27,30] |
| $1/\nu$ | Mean duration of antibiotic treatment | 8 days | [15,31] |
| $\mu$ | Birth rate | $12.6 \times 10^{-3}\ \text{yr}^{-1}$ | INSEE |
| $m$ | Death rate | $8.6 \times 10^{-3}\ \text{yr}^{-1}$ | INSEE |
| $N(0)$ | Initial population size | $59.267 \times 10^{6}$ | INSEE |
| $c(0)$ | Initial carriage prevalence | 0.3 | [32] |
| $c_V(0)$ | Initial frequency of V-serotype carriers | 0.53 | NRCP 2001 data |
| $r_V(0)$ | Initial resistance frequency in V-serotypes | 0.74 | NRCP 2001 data |
| $r_{NV}(0)$ | Initial resistance frequency in NV-serotypes | 0.29 | NRCP 2001 data |
| $\phi, \psi$ | Notification probabilities to NRCP (odd years, even years) | 0.65, 0.53 | Text S1.2 |
| $\tau(t)$ | Per capita antibiotic-exposure rate | Fig. 1 | NHI |

| | | | |
|---|---|---|---|
| | | | reimbursement data |
| v($t$) | Vaccine coverage | Fig. 1 | 1/97 sample from NHI |
| $\beta_X$ | Mean transmission rate for strain X (yr$^{-1}$) | Estimated | — |
| $\rho_X$ | Mean infection rate for strain X (yr$^{-1}$) | Estimated | — |
| $\rho$ | Amplitude of infection rate seasonality | Estimated | — |
| $\theta$ | Between-strain competition | Estimated | — |
| $\sigma_V$ | PCV7 efficacy on carriage acquisition | Estimated | — |

Parameter unit, if any, is indicated between parentheses. Subscripts S and R refer to susceptible and resistant strains, respectively. Abbreviations: INSEE, National Institute of Statistics and Economic Studies; NRCP, French National Reference Center for Pneumococci; NHI, National Health Insurance system.

**Table S5. Parameter estimates and comparison of the VNV and SR models.** See Table S4 for a description of the model parameters.

| VNV model | | SR model | |
|---|---|---|---|
| Quantity | Estimate (99% CI) | Quantity | Estimate (99% CI) |
| $\beta_V$ ($yr^{-1}$) | 14.7 (14.4–15.4) | $\beta_S$ ($yr^{-1}$) | 15.2 (14.5–15.7) |
| $\beta_{NV}/\beta_V$ | 1.00 (0.99–1.01) | $\beta_R/\beta_S$ | 0.954 (0.950–0.957) |
| $\rho_V$ ($\times10^{-5}$ $yr^{-1}$) | 3.4 (3.3–4.2) | $\rho_S$ ($\times10^{-5}$ $yr^{-1}$) | 3.8 (3.5–4.8) |
| $\rho_{NV}/\rho_V$ | 1.01 (0.81–1.26) | $\rho_R/\rho_S$ | 0.78 (0.54–0.93) |
| $\rho$ | 0.57 (0.52–0.63) | $\rho$ | 0.57 (0.51–0.63) |
| $\theta$ | 0.42 (0–1) | $\theta$ | 0.82 (0.60–1) |
| $\sigma_V$ | 0.78 (0.47–1) | $\sigma_V$ | 0.95 (0.87–1) |
| $\sigma_S$ | 0.82 (0–1) | $\sigma_S$ | — |
| $\sigma_R$ | 0.10 (0–1) | $\sigma_R$ | — |
| $\phi_S$ | 0.75 (0–1) | $\phi_S$ | — |
| $\phi_R$ | 3.0 (0–6.1) | $\phi_R$ | — |
| *L* | −932.6 | *L* | −925.6 |
| No parameters* | 11 | No parameters | 7 |
| AIC | 1887.2 | AIC | 1865.2 |
| ΔAIC | 22.0 | ΔAIC | 0 |

Parameter unit, if any, is indicated between parentheses. *We were unable to fit the VNV model with 7 estimated parameters. Therefore, we estimated 4 more parameters (antibiotic-related parameters) for this model. Notably, the estimates of $\sigma_S$ and $\sigma_R$ were unrealistic in this case ($\sigma_S$>

$\sigma_R$), thus reinforcing our main conclusion that differences of fitness between penicillin-susceptible and penicillin-resistant strains are required to explain the data.

**Table S6. Sensitivity analyses: parameter estimates when varying the values of fixed model parameters.**

| Pair tested | Range tested | $\beta_R/\beta_S$ (range) | $\rho_R/\rho_S$ (range) |
|---|---|---|---|
| $\sigma_S$, $\sigma_R$ | (0−0.5)×(0.5−1) | (0.93−0.98) | (0.68−0.84) |
| $\phi_S$, $\phi_R$ | (0−1)×(1−5) | (0.92−0.98) | (0.64−0.87) |
| $\sigma_R$, $\phi_R$ | (0.5−1)×(1−5) | (0.90−0.98) | (0.67−0.88) |
| $1/\omega$ (days), $1/\nu$ (days) | (1−8)×(5−15) | (0.92−0.98) | (0.65−0.90) |
| $c_0$, $\gamma$ (day$^{-1}$) | (0.15−0.4)×(5−20) | (0.91−0.96) | (0.67−0.90) |

Parameter units, if any, are indicated between parentheses.

**Table S7. Sensitivity analyses: parameter estimates after removing one serotype from the data.** We identified the 3 most prevalent vaccine serotypes (19F, 14, and 23F) and nonvaccine serotypes (19A, 3, and 7F); for each of these serotypes, we created a new data set by removing this serotype's data and performed the estimations as before. In each case, we performed 200 replicate estimations with random starting values for the estimated parameters; in the table, we report the mean value (range) of the 10 best estimates (i.e., numbers between parentheses do not correspond to 99% confidence intervals).

| Serotype | V/NV | Transmissibility ratio R/S | Invasiveness ratio R/S |
|---|---|---|---|
| 19F | V | 0.96 (0.95–0.96) | 0.47 (0.30–0.74) |
| 14 | V | 0.95 (0.95–0.96) | 0.50 (0.23–0.91) |
| 23F | V | 0.95 (0.95–0.96) | 0.46 (0.34–0.73) |
| 19A | NV | 0.95 (0.95–0.96) | 0.76 (0.47–0.95) |
| 3 | NV | 0.95 (0.95–0.96) | 0.72 (0.5–1.2) |
| 7F | NV | 0.95 (0.95–0.96) | 1.1 (0.75–1.50) |

## References


1 Bogaert, D., De Groot, R. & Hermans, P. W. *Streptococcus pneumoniae* colonisation: the key to pneumococcal disease. *Lancet Infect. Dis.* **4**, 144–154, doi:10.1016/S1473-3099(04)00938-7 (2004).

2 Lipsitch, M. *et al.* Competition among *Streptococcus pneumoniae* for intranasal colonization in a mouse model. *Vaccine* **18**, 2895–2901 (2000).

3 Erasto, P. *et al.* Modelling multi-type transmission of pneumococcal carriage in Bangladeshi families. *Epidemiol. Infect.* **138**, 861–872, doi:10.1017/S0950268809991415 (2010).

4 Granat, S. M. *et al.* Epidemiological evidence for serotype-independent acquired immunity to pneumococcal carriage. *J. Infect. Dis.* **200**, 99–106, doi:10.1086/599364 (2009).

5 Dowell, S. F., Whitney, C. G., Wright, C., Rose, C. E., Jr. & Schuchat, A. Seasonal patterns of invasive pneumococcal disease. *Emerg. Infect. Dis.* **9**, 573–579 (2003).

6 Gray, B. M., Turner, M. E. & Dillon, H. C., Jr. Epidemiologic studies of *Streptococcus pneumoniae* in infants. The effects of season and age on pneumococcal acquisition and carriage in the first 24 months of life. *Am. J. Epidemiol.* **116**, 692–703 (1982).

7 Hussain, M. *et al.* A longitudinal household study of *Streptococcus pneumoniae* nasopharyngeal carriage in a UK setting. *Epidemiol. Infect.* **133**, 891–898, doi:10.1017/S0950268805004012 (2005).

8 Sutton, K. L., Banks, H. T. & Castillo-Chavez, C. Estimation of invasive pneumococcal disease dynamics parameters and the impact of conjugate vaccination in Australia. *Math Biosci Eng* **5**, 175–204 (2008).

9 Varon, E. & Gutmann, L. Rapport d'activité 2002. (Centre National de Référence des Pneumocoques, Paris, 2002).

10 Black, S. *et al.* Efficacy, safety and immunogenicity of heptavalent pneumococcal conjugate vaccine in children. Northern California Kaiser Permanente Vaccine Study Center Group. *Pediatr. Infect. Dis. J.* **19**, 187–195 (2000).

11 Rinta-Kokko, H., Dagan, R., Givon-Lavi, N. & Auranen, K. Estimation of vaccine efficacy against acquisition of pneumococcal carriage. *Vaccine* **27**, 3831–3837, doi:10.1016/j.vaccine.2009.04.009 (2009).

12 Sabuncu, E. *et al.* Significant reduction of antibiotic use in the community after a nationwide campaign in France, 2002-2007. *PLoS Med.* **6**, e1000084, doi:10.1371/journal.pmed.1000084 (2009).

13 Lipsitch, M. & Samore, M. H. Antimicrobial use and antimicrobial resistance: a population perspective. *Emerg. Infect. Dis.* **8**, 347–354, doi:10.3201/eid0804.010312 (2002).

14 Ghaffar, F. *et al.* Effects of amoxicillin/clavulanate or azithromycin on nasopharyngeal carriage of *Streptococcus pneumoniae* and *Haemophilus influenzae* in children with acute otitis media. *Clin. Infect. Dis.* **31**, 875–880, doi:10.1086/318160 (2000).

15 Varon, E. *et al.* Impact of antimicrobial therapy on nasopharyngeal carriage of *Streptococcus pneumoniae*, *Haemophilus influenzae*, and *Branhamella catarrhalis* in children with respiratory tract infections. *Clin. Infect. Dis.* **31**, 477–481, doi:10.1086/313981 (2000).

16 Lipsitch, M. Measuring and interpreting associations between antibiotic use and penicillin resistance in *Streptococcus pneumoniae*. *Clin. Infect. Dis.* **32**, 1044–1054, doi:10.1086/319604 (2001).

17 Dagan, R., Barkai, G., Leibovitz, E., Dreifuss, E. & Greenberg, D. Will reduction of antibiotic use reduce antibiotic resistance?: The pneumococcus paradigm. *Pediatr. Infect. Dis. J.* **25**, 981–986, doi:10.1097/01.inf.0000239266.20642.26 (2006).

18 Samore, M. H. *et al.* Mechanisms by which antibiotics promote dissemination of resistant pneumococci in human populations. *Am. J. Epidemiol.* **163**, 160–170, doi:10.1093/aje/kwj021 (2006).

19 Ionides, E. L., Breto, C. & King, A. A. Inference for nonlinear dynamical systems. *Proc. Natl. Acad. Sci. USA* **103**, 18438–18443, doi:10.1073/pnas.0603181103 (2006).

20 Auranen, K., Mehtala, J., Tanskanen, A. & M, S. K. Between-strain competition in acquisition and clearance of pneumococcal carriage--epidemiologic evidence from a longitudinal study of day-care children. *Am. J. Epidemiol.* **171**, 169–176, doi:10.1093/aje/kwp351 (2010).

21 Mehtala, J., Antonio, M., Kaltoft, M. S., O'Brien, K. L. & Auranen, K. Competition between *Streptococcus pneumoniae* strains: implications for vaccine-induced replacement in colonization and disease. *Epidemiology* **24**, 522–529, doi:10.1097/EDE.0b013e318294be89 (2013).

22 Scherer, A. & McLean, A. Mathematical models of vaccination. *Br. Med. Bull.* **62**, 187–199 (2002).

23 Maher, M. C. *et al.* The fitness cost of antibiotic resistance in *Streptococcus pneumoniae*: insight from the field. *PLoS One* **7**, e29407, doi:10.1371/journal.pone.0029407 (2012).

24 Levy, C. *et al.* Pneumococcal meningitis in French children before and after the introduction of pneumococcal conjugate vaccine. *Pediatr. Infect. Dis. J.* **30**, 168–170 (2011).

25 Cohen, R. *et al.* Dynamic of pneumococcal nasopharyngeal carriage in children with acute otitis media following PCV7 introduction in France. *Vaccine* **28**, 6114–6121, doi:10.1016/j.vaccine.2009.05.037 (2010).

26 Domenech de Celles, M. *et al.* Intrinsic epidemicity of *Streptococcus pneumoniae* depends on strain serotype and antibiotic susceptibility pattern. *Antimicrob. Agents Chemother.* **55**, 5255–5261, doi:10.1128/AAC.00249-11 (2011).

27 Opatowski, L. *et al.* Antibiotic innovation may contribute to slowing the dissemination of multiresistant *Streptococcus pneumoniae*: the example of ketolides. *PLoS One* **3**, e2089, doi:10.1371/journal.pone.0002089 (2008).

28 Mossong, J. *et al.* Social contacts and mixing patterns relevant to the spread of infectious diseases. *PLoS Med.* **5**, e74, doi:10.1371/journal.pmed.0050074 (2008).

29 Hogberg, L. *et al.* Age- and serogroup-related differences in observed durations of nasopharyngeal carriage of penicillin-resistant pneumococci. *J. Clin. Microbiol.* **45**, 948–952, doi:10.1128/JCM.01913-06 (2007).

30 Temime, L., Boelle, P. Y., Courvalin, P. & Guillemot, D. Bacterial resistance to penicillin G by decreased affinity of penicillin-binding proteins: a mathematical model. *Emerg. Infect. Dis.* **9**, 411–417, doi:10.3201/eid0904.020213 (2003).

31 Guillemot, D. *et al.* Inappropriateness and variability of antibiotic prescription among French office-based physicians. *J. Clin. Epidemiol.* **51**, 61–68 (1998).

32 Flasche, S. *et al.* Effect of pneumococcal conjugate vaccination on serotype-specific carriage and invasive disease in England: a cross-sectional study. *PLoS Med.* **8**, e1001017, doi:10.1371/journal.pmed.1001017 (2011).
